# The Relationship of Circulating Choline and Choline-Related Metabolite Levels with Health Outcomes: A Scoping Review of Genome-Wide Association Studies and Mendelian Randomization Studies

**DOI:** 10.1016/j.advnut.2023.100164

**Published:** 2023-12-20

**Authors:** Lauren E Louck, Kelly C Cara, Kevin Klatt, Taylor C Wallace, Mei Chung

**Affiliations:** 1 Friedman School of Nutrition Science and Policy, Tufts University, Boston, MA, United States; 2 Nutritional Sciences and Toxicology, University of California, Berkeley, CA, United States; 3 Think Health Group, Inc, Washington, DC, United States

**Keywords:** choline, glycerophosphocholine, lysophosphatidylcholines, phosphatidylcholines, sphingomyelins, betaine, trimethylamine N-oxide, Mendelian randomization, genome-wide association study, scoping review

## Abstract

Choline is essential for proper liver, muscle, brain, lipid metabolism, cellular membrane composition, and repair. Understanding genetic determinants of circulating choline metabolites can help identify new determinants of choline metabolism, requirements, and their link to disease endpoints. We conducted a scoping review to identify studies assessing the association of genetic polymorphisms on circulating choline and choline-related metabolite concentrations and subsequent associations with health outcomes. This study follows the Preferred Reporting Items for Systematic Reviews and Meta-Analyses statement scoping review extension. Literature was searched to September 28, 2022, in 4 databases: Embase, MEDLINE, Web of Science, and the Biological Science Index. Studies of any duration in humans were considered. Any genome-wide association study (GWAS) investigating genetic variant associations with circulating choline and/or choline-related metabolites and any Mendelian randomization (MR) study investigating the association of genetically predicted circulating choline and/or choline-related metabolites with any health outcome were considered. Qualitative evidence is presented in summary tables. From 1248 total reviewed articles, 53 were included (GWAS = 27; MR = 26). Forty-two circulating choline-related metabolites were tested in association with genetic variants in GWAS studies, primarily trimethylamine N-oxide, betaine, sphingomyelins, lysophosphatidylcholines, and phosphatidylcholines. MR studies investigated associations between 52 total unique choline metabolites and 66 unique health outcomes. Of these, 47 significant associations were reported between 16 metabolites (primarily choline, lysophosphatidylcholines, phosphatidylcholines, betaine, and sphingomyelins) and 27 health outcomes including cancer, cardiovascular, metabolic, bone, and brain-related outcomes. Some articles reported significant associations between multiple choline types and the same health outcome. Genetically predicted circulating choline and choline-related metabolite concentrations are associated with a wide variety of health outcomes. Further research is needed to assess how genetic variability influences choline metabolism and whether individuals with lower genetically predicted circulating choline and choline-related metabolite concentrations would benefit from a dietary intervention or supplementation.

**Statements of significance**

To the best of our knowledge, no article to date has reviewed and summarized the current literature regarding genetically predicted circulating choline and choline-related metabolites. Additionally, this scoping review summarizes the research reporting disease endpoints and health outcomes associated with genetically predicted circulating choline and choline-related metabolites.

## Introduction

Choline is an essential nutrient with defined Dietary Reference Intake values in the United States and Canada since 1998 [1]. It is essential for proper liver, muscle, and brain functions, lipid metabolism, and cellular membrane composition and repair [[1], [2], [3], [4]]. Choline depletion/repletion studies have linked choline deficiency with liver dysfunction and muscle damage [5,6]. Choline intake and adequate fetal choline supply are particularly critical during development, influencing the risk of neural tube defects [[7], [8], [9]], modifying septo-hippocampal circuitry and brain function [10], as well as postnatal cognition and chronic disease risk factors [11]. Higher choline intake in adults has been linked to better cognitive performance and brain morphology [12]. Data from the NHANES show estimated choline intakes to be slightly above 300 mg/d for nonpregnant, nonlactating individuals [[13], [14], [15]]. A large percentage of individuals are not consuming the Adequate Intake (AI) for choline, with a mere 10% of Americans and 8% of pregnant women currently meeting their gender- and life-stage specific AI for choline [13]. Recently, researchers highlighted the difficulty of meeting the AI for choline without egg intake or supplementation [13]. Choline in the diet is absorbed and can be partitioned postconsumption toward several different metabolic fates and is present in circulation as free choline or as metabolites [e.g., betaine, phosphatidylcholines (PCs), sphingomyelins (SMs), and acetylcholine); unabsorbed choline can be scavenged by the microbiota and metabolized to trimethylamine (TMA), which is absorbed and oxidized to TMA N-oxide (TMAO). Circulating choline-related metabolite concentrations are the most readily accessible for clinical assessment but are often challenging to interpret, as they represent an aggregate of dietary inputs, endogenous synthesis, and various concentrations of homeostatic regulation across absorption, tissue-level distribution, metabolism, and excretion.

Although these numerous inputs add complexity to the interpretation of circulating choline-related metabolites, such complexity lends itself to discovery. Genetic variants associated with circulating choline-related metabolites identified in Genome-Wide Association Studies (GWAS) have the potential to identify novel determinants of choline’s metabolic handling as well as potential candidates for gene-x-diet interactions (i.e., nutrigenetics). GWAS facilitates the identification of novel regulators of whole-body and cellular choline metabolism beyond the extensive literature of candidate gene approaches interrogating common variants throughout one-carbon metabolism on choline requirements and metabolism [16]. Recently, the capacity to assess genetic determinants of circulating metabolites has become possible because of the increase in large cohort studies with both high-throughput DNA sequencing data as well as untargeted metabolomic approaches. However, a targeted examination of these analyses’ results as they relate specifically to choline has not been undertaken.

Understanding genetic determinants of circulating choline-related metabolites is not only beneficial for identifying new determinants of choline metabolism and requirements but also for linking choline metabolism to disease endpoints. Indeed, such variants may be used in instrumental variable (IV) analyses [i.e., Mendelian Randomization (MR)] to identify possible links between circulating choline-related metabolites and disease endpoints [[17], [18], [19]]. Although these links do not immediately infer causality, they may provide more robust and reliable associations, as they serve as proxies of lifetime exposure and are less prone to confounding and reverse causation when the appropriate IV is used [20]. However, until recently, the lack of genetic variants associated with circulating choline-related metabolites has hampered the use of MR to link choline metabolism to potential disease endpoints.

Thus, we have undertaken a scoping review of genetic epidemiological studies, namely GWAS and MR approaches, that have captured genetic data or utilized genetic IVs, measured circulating choline and choline-related metabolites, and examined associations with any health outcomes. The results of this review summarize the current literature regarding metabolic-x-genetic interactions that influence choline metabolism and the relationship of circulating choline to health outcomes.

## Methods

A scoping review was conducted and followed the Preferred Reporting Items for Systematic Reviews and Meta-Analyses statement scoping review extension [21,22]. The PICO (population, interest, context, and outcome) framework was used to search and identify relevant literature [23,24].

### Literature search and selection

Literature was searched to September 28, 2022, in 4 databases: Embase, Ovid MEDLINE, Web of Science (all databases), and the Biological Science Index. Searches included key terms related to circulating choline and choline-related metabolites, as well as GWAS and MR study designs. Results were limited to human studies of any duration. The complete search strategy for MEDLINE is presented in the supplement. The database search was supplemented with 3 additional searches for reports published up to December 2022. First, articles were hand-selected for screening based on expert knowledge. Second, references were mined from articles suspected of reporting on genetic-related choline/choline metabolite data in supplemental files. Third, the National Human Genome Research Institute-European Bioinformatics GWAS Catalogue [25] was searched for choline-related studies and single nucleotide polymorphisms. The supplemental files of these articles were double-screened and selected for inclusion based on the screening criteria and selection process. Any GWAS investigating genetic variant associations with circulating choline and/or choline/related metabolites and any MR study investigating the effect of circulating choline and/or choline-related metabolites on any health outcome were considered.

Duplicate articles were removed in EndNote (Clarivate Analytics) version X9 before screening. Titles and abstracts were screened by 2 independent investigators using the Rayyan web (Rayyan Systems, Inc.) application for systematic reviews [26]. Full-text documents were screened by 1 investigator, and exclusions were double-screened by a second independent investigator. Conflicts were resolved by discussion between the 2 investigators. The complete eligibility criteria used during screening are presented in Table 1.

**TABLE 1:** Eligibility criteria for inclusion in the review

Category	Inclusion criteria	Exclusion criteria
Study design	Mendelian randomization (MR) studies Genetic instrumental variable analysis studies Genome-wide Association Study (GWAS or GWA study) Whole Genome Association Study (WGAS or WGA study)	Any reviews or meta-analyses Case-cohort studies Case reports or case series Cohort studies Conference proceedings or abstracts cross-sectional studies Letters to the editor Randomized or nonrandomized controlled trials Uncontrolled trials
Study duration	No restriction	None
Sample size	No restriction	None
Intervention/exposure	For GWAS studies: Single nucleotide polymorphisms (SNPs) For MR studies: 1. Any instrumental variable with SNPs associated with choline/choline metabolites or 2. Serum or plasma choline or choline-related metabolites. Choline/choline metabolites of interest include: • Choline, bursine, vidine, or fagine • Acetylcholine • Betaine • Choline bitartrate • Glycerolphosphocholine • Glycerophosphocholine • Glycerophosphorylcholine • Glycerylphosphorylcholine • Lecithins • Lysophosphatidylcholine • Phosphatidylcholines • Phosphocholine • Sphingomyelins • Trimethylamine • Trimethylamine N-oxide (TMAO)	GWAS studies: No SNPs MR studies: No choline/choline metabolites or associated SNPs.
Comparator	No restriction	None
Outcomes	For GWAS studies: Serum or plasma choline or choline-related metabolites including: • Choline, bursine, vidine, or fagine • Acetylcholine • Betaine • Choline bitartrate • Glycerolphosphocholine • Glycerophosphocholine • Glycerophosphorylcholine • Glycerylphosphorylcholine • Lecithins • Lysophosphatidylcholine • Phosphatidylcholines • Phosphocholine • Sphingomyelins • Trimethylamine • TMAO For MR studies: Any health outcome (e.g., any disease, health measures like blood pressure, etc.)	GWAS studies: No choline/choline metabolites. MR studies: No health outcomes
Date of publication	No restriction	None
Publication status	Peer-reviewed publications	Publications that are not peer-reviewed
Language of publication	No restriction	None
Country	No restriction	None
Study participants	Humans	Animals
Age of study participants	No restriction	None
Health status of participants	No restriction	None

### Data extraction

Data were extracted using an internally created form. Data fields include author, year, title, funding source, primary study aim, study design, cohort/data source, cohort ethnicity, sample size, cohort age, percent female, relatedness, choline/choline metabolite type, and health outcome(s) assessed. Quantitative results were separately extracted only for MR studies and only if the result was statistically significant according to the study-reported significance threshold. Extracted results included the MR method, exposure, outcome, effect size, 95% confidence interval, and *P* value.

### Data reporting and synthesis

Study characteristics and qualitative data are presented in separate tables for GWAS and MR studies to facilitate narrative synthesis. A summary of health outcomes found to be significantly associated with choline or choline-related metabolites across included studies was reported in tables, and a heatmap was used to highlight common findings. Summary data were calculated using Microsoft Excel version 16.70.

## Results

**FIGURE 1:**
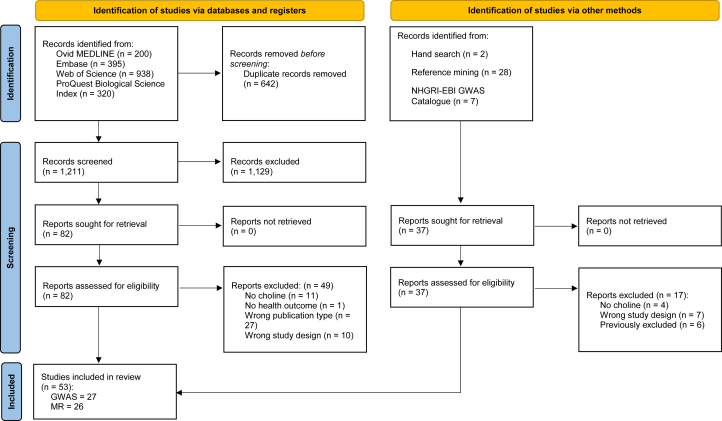
PRISMA 2020 flow diagram for new systematic reviews, which included searches of databases, registers, and other sources. GWAS, Genome-Wide Association Study; MR, Mendelian randomization; PRISMA, Preferred Reporting Items for Systematic Reviews and Meta-Analyses. Reproduced from: M.J. Page, J.E. McKenzie, P.M. Bossuyt, I. Boutron, T.C. Hoffmann, C.D. Mulrow, et al., The PRISMA 2020 statement: an updated guideline for reporting systematic reviews, BMJ. 372 (2021) n71, https://doi.org/10.1136/bmj.n71. For more information, visit http://www.prisma-statement.org/.

Out of 1248 total reviewed articles, 53 met the inclusion criteria (GWAS = 27; MR = 26). Figure 1 outlines the study search and selection process. A list of excluded full-text articles and reasons for exclusion is presented in Supplementary Table 1. The results for GWAS and MR studies are presented in separate sections below.

### GWAS

The 27 included GWAS studies utilized data from 64 total data sources representing 33 unique cohorts. The majority of these cohorts included Europeans (76%), whereas the others represented transethnic populations (12%), African Americans (6%), and individuals from Australia, Qatar, or South Korea (1 cohort each). Of the unique cohorts, 58% included unrelated individuals, 39% included related individuals (e.g., families, siblings, twins, or spouses), and for 1 cohort, relatedness was not clear. In 4 of the GWAS studies, related individuals were excluded, or relatedness was otherwise accounted for in the analyses. The reported mean age of participants was 20 y or greater for all data sources; the highest reported participant age was 81 y. Age was not reported for 10 data sources from 7 of the included studies. In the 64 reported data sources, the study population size ranged from 91 to 898,130 participants, where 34% of reported data sources had fewer than 1000 participants, 50% of data sources had 1000 to 5000 participants, and the remaining 16% of data sources had >5000 participants.

From the 27 included GWAS articles, 42 unique circulating choline metabolites were tested in association with genetic variants [primarily TMAO, betaine, SMs, lysophosphatidylcholines (LysoPCs), and PCs]. A full list of choline metabolites and key findings reported in the individual GWAS are presented in Table 2 [[27], [28], [29], [30], [31], [32], [33], [34], [35], [36], [37], [38], [39], [40], [41], [42], [43], [44], [45], [46], [47], [48], [49], [50], [51], [52], [53]].

**TABLE 2:** Individual study characteristics for included Genome-Wide Association Studies (Single nucleotide polymorphism-x- metabolite association) studies

Authors, y (reference)	Choline type(s) reported	Outcome	Cohort or data source	Sample size (% female)	Mean age or range, y	Ethnicity	Relatedness	Key main text findings
Al-Khelaifi, 2019 [27]	Arachidonoylcholine stearoylcholine, betaine, choline phosphate, glycerophosphorylcholine (GPC), linoleoylcholine, oleoylcholine, choline, palmitoylcholine, palmitoloelycholine, PC, SMs	Genetic variation in metabolic individuality of endurance athletes.	Elite athletes cohort	490 (13.5)	NR	Transethnic	None	Multiple SNPs were significantly associated with circulating choline, betaine, GPC, PCs, and SMs.
Aslibekyan, 2017 [28]	TMAO	Heritable determinants of plasma TMAO	Genetics of Lipid Lowering Drugs and Diet Network (GOLDN)	944 (48.83)	∼48.75	European	Families, siblings	The genome-wide study yielded 1 significant variant association with circulating TMAO at the genome-wide level, located in an intergenic region on chromosome 4.
Demirkan, 2015 [29]	TMAO/betaine, betaine	Novel gene-metabolite associations	Erasmus Rucphen Family (ERF)	2415 (∼57)	50.36	Dutch	Families	Exome variants were not significantly associated with either circulating TMAO/betaine or betaine.
Draisma, 2015 [30]	Hydroxylacyloylsphingosylphosphocholine, LysoPCs PCs, SMs	Variation in human blood metabolite concentrations	Netherlands Twin Register (NTR)	1196 (32.8%)	53.09	Netherlands	Twins	Genetic variants at the genome-wide level were associated with multiple forms of circulating LysoPCs, PCs, and SMs.
			Queensland Institute of Medical Research(QIMR) cohort	848 (48.9%)	20.17	Australia	None	
			Twins United Kingdom (TwinsUK)	1235 (96.4%)	57.95	United Kingdom	Twins	
			Leiden Longevity Study (LLS)	657 (50.4%)	62.96	Netherlands	Families	
			Estonian Genome Center of University of Tartu Cohort (EGCUT)	872 (38.8%)	37.4	Estoria	None	
			Cooperative Health Research in the Region Augsburg F4 (KORA F4)	1797 (51.1%)	60.89	Germany	None	
			Erasmus Rucphen Family (ERF) Study	940 (56.2%)	48.30	Netherlands	Families	
			Cooperative Health Research in the Region Augsburg (KORA)	1,182 (52.2%)	49.02	Germany	None	
Gieger, 2008 [31]	SMs, PCs	Genetic variants associated with 363 circulating metabolites	Cooperative Health Research in the Region Augsburg S3 (KORA S3)	284 (0)	35–79	German	None	rs174548 in the F*ADS1* gene was significantly associated with circulating PCs and SMs.
Hartiala, 2014 [32]	TMAO	Genetic factors associated with plasma TMAO	Cleveland Clinic GeneBank study	3865 (30.4%)	64	Non-Caucasian or Hispanic (based on eligibility criteria from https://clinicaltrials.gov/ct2/show/NCT00590200)	None	The genome-wide association for plasma identified 2 loci with suggestive evidence of association on chromosomes 1q23.3 and 2p12 with plasma TMAO concentrations. However, genotyping of the lead variants at these loci in replication subjects did not show an association with plasma TMAO concentrations.
Hartiala, 2016 [33]	Betaine	Genetic factors associated with plasma betaine and CAD	Cleveland Clinic GeneBank study	8668 (29.7%)	64	Non-Caucasian or Hispanic (based on eligibility criteria from https://clinicaltrials.gov/ct2/show/NCT00590200)	None	2 loci were significantly associated with circulating betaine on chromosomes 2q34 and 5q14.1.
Hicks, 2009 [34]	SM, dihydro-SM	Genetic variants associated with circulating (SM) and dihydro-SM concentration	Northern Swedish Population Health Study (NSPHS)	656 (NR)	NR	Swedish	Families	Genetic variants across 23 candidate genes involved in sphingolipid-metabolizing pathways were significantly associated with circulating SM and dihydro-SM.
Hysi, 2022 [35]	Arachidonoylcholine, betaine, choline, GPCs, PC, SMs	Genomic regions associated with circulating metabolite concentrations.	National Institute for Health Research Bioresource cohort (NIHR UK)	8809 (NR)	NR	European	Unsure	A SNP in the *MYRF* gene was strongly associated with circulating Arachidonoylcholine. Multiple SNPs were associated with circulating betaine, GPCs, and SMs.
			Cooperative Health Research in the Region of Augsburg F4 (KORA F4)	1768 (NR)	NR	German	None	
Illig, 2010 [36]	LysoPCs, PCs, SMs	Cardiovascular and metabolic disease-related SNP associations with metabolites	Cooperative Health Research in the Region of Augsburg S4 (KORA S4)	1809 (∼52.1)	32–81	German	None	Top cardiovascular and metabolic disease-related SNPs were significantly associated with numerous circulating LysoPCs, PCs, and SMs.
			Twins United Kingdom (TwinsUK)	422 (NR)	NR	British	The authors selected unrelated individuals for analysis	
Kettunen, 2016 [37]	SMs, PCs	SNPs associated with circulating cardiovascular disease risk metabolites, focusing on the LPA locus.	Estonian Genome Center of University of Tartu Cohort (EGCUT)	3287 (46.3)	58	Estonian	None	No significant associations were found between SNPs and circulating PCs or SMs.
			Erasmus Rucphen Family Study (ERF)	2118 (48.2)	58	Dutch	Families	
			Finnish Twin Cohort(FTC)	664 (23.9)	50		Twins	
			Dietary, Lifestyle, and Genetic determinants of Obesity and Metabolic syndrome study [DILGOM (FR97, a subsample of Finnish National study (FINRISK) 1997)]	3661 (45.3)	55	Finnish	None	
			Genetic Predisposition of Coronary Heart Disease in Patients Verified with Coronary Angiogram(COROGENE)	828 (53.2)	54	Finnish	None	
			Genetics of METabolic Syndrome (GenMets)	572 (55.8)	57	Finnish	None	
			Helsinki Birth Cohort Study (HBCS)	708 (61.3)	60	Finnish	None	
			Cooperative Health Research in the Region of Augsburg (KORA)	1745 (60.9)	52	German	None	
			Leiden Longevity Study (LLS)	2227 (59.2)	54	Dutch	Families	
			Netherlands Twin Register (NTR)	1192 (38.8)	64	Dutch	None	
			Northern Finland Birth Cohort 1966 (NFBC 1966)	4709 (31.2)	51	Finnish	None	
			Finnish National study (FINRISK) subsample of incident cardiovascular cases and controls (Personalized Responses to Dietary Composition Trial-Cardiovascular Disease [PREDICT-CVD])	374 (47.5)	37	Finnish	None	
			Estonian Genome Center of University of Tartu Cohort (EGCUT sub-cohort, PROTE)	597 (38.3)	51	Estonian	None	
			The Cardiovascular Risk in Young Finns Study (YFS)	2390 (37.7)	54	Finnish	None	
Kettunen, 2012 [38]	Choline, PC	SNP associations with 216 serum metabolites	Northern Finland Birth Cohort 1966 (NFBC1966)	4703 (31.0)	51	Finnish	None	Multiple SNPs were significantly associated with circulating choline and PC.
			The Cardiovascular Risk in Young Finns Study (YFS)	1904 (37.7)	54	Finnish	None	
			Helsinki Birth Cohort Study (HBCS)	708 (61.3)	60	Finnish	None	
			Genetics of METabolic Syndrome (GenMets) subsample of Finnish Health2000 examination survey	572 (55.8)	57	Finnish	None	
			The Dietary, Lifestyle, and Genetic determinants of Obesity and Metabolic Syndrome (DILGOM)	443 (50.1)	56	Finnish	None	
			Finnish Twin cohorts 12 and 16 (FinnTwin12 and FinnTwin16)	1269 (23.9)	55	Finnish	None	
Lee, 2016 [39]	LysoPC	Genetic variants associated with metabolomics identified T2D-related metabolites	Korea Association Resource Survey 2 (KARE S2) cohort	2240 (∼55.2%)	∼56	South Korean	None	Genetic variants were not associated with circulating LysoPC.
Li, 2018 [40]	LysoPC a C20:4, PC ae C38:4, PC ae C38:5, PC ae C36:5, PC aa C36:4, PC aa C36, PC aa C28:1, PC ae C32:1, SM (OH) C14:1	Genetic variants of the concentrations of 139 serum metabolites and 41 urine metabolites in people with chronic kidney disease.	German Chronic Kidney Disease (GCKD) Study	1143 (37%)	56	German	None	One genetic variant at *TMEM258* was significantly associated with LysoPCs. Eight genetic variants at MYRF, *TMEM258, FADS1, FADS2, SGPP1*, *SYNE2, and TMEM229B* were significantly associated with circulating PCs. One genetic variant at *SGPP1* and *SYNE2* was significantly associated with circulating SMs.
Li-Gao, 2021 [41]	PCs and other cholines, SMs, and total choline	Genetics and heritability of plasma metabolomic measures	Netherlands Epidemiology of Obesity (NEO) study	6671 [5705 analyzed (47.49)]	45–65	Dutch	None	Multiple SNPs were significantly associated with circulating PCs and other cholines, SMs, and total choline.
Long, 2017 [42]	Betaine, choline, PCs, SMs	Abnormal blood metabolic phenotypes	Twins United Kingdom (TwinsUK)	1960 (96.6%)	Median = 58, range = 32–87	European descent	Twins	SNPs were not associated with circulating betaine or choline. Multiple SNPs were significantly associated with multiple forms of circulating PCs and SMs.
Richardson, 2022 [43]	Cholines, SMs	Lipid-modifying therapeutic targets on the blood metabolome and subsequent CVD risk reduction.	UKBiobank	115,082	40–71	European	None	Multiple SNPs were significantly associated with circulating cholines and SMs.
			Coronary Artery Disease Genome-Wide Replication and Meta-analysis (CARDIoGRAM) plus the Coronary Artery Disease Genetics (CARDIoGRAMplusC4D) Consortium	CARDIoGRAMplusC4D consortium	60,801 CAD cases (NR); 123,504 controls (NR)	NR	Transethnic	
			Diabetes Meta-Analysis of Trans-Ethnic Association Studies (DIAMANTE) Consortium	DIAMANTE consortium	74,124 T2D cases (NR); 824,006 controls (NR)	NR	European	
Rhee, 2013 [44]	PCs, LysoPCs, SMs	Relative contributions of inherited factors on the plasma metabolome	Framingham Heart Study (FHS)	2076 (51%)	55	European descent	Spouses	SNPs in the FADS1-3 locus were significantly associated with multiple circulating PCs and LysoPCs. One SNP in the GCKR locus was associated with circulating PC 34:3 and PC 32:2. SNPs in the SYNE2 and PDE4D genes were associated with circulating SM 14:0 and 24:1, respectively.
Rhee, 2016 [45]	Cholines, PCs, SMs	Genetic variants associated with the plasma metabolome	Framingham Heart Study (FHS)	2076 (51%)	55	European descent	Spouses	No genetic variants were significantly associated with circulating choline metabolites.
			Atherosclerosis Risk in Communities (ARIC) Study	1528 (NR)	Range = 45–64	European descent	None	
Shin, 2014 [46]	Betaine, palmitoyl SM	Genetic loci influencing the human metabolome	Twins United Kingdom (TwinsUK)	6056 (93%)	53	British	Twins	SNPs in CBS, CPS1, and BHMT (rs16876394) loci were significantly associated with circulating betaine. One SNP in the SPTLC3 locus was significantly associated with circulating palmitoyl-SM.
			Cooperative Health Research in the Region of Augsburg (KORA) study (F4 and S4)	1768 (50%)	61	German	None	
Surendran, 2022 [47]	Choline, choline phosphate, GPC, PC, SMs	Genetic architecture of over 900 metabolites	Efficiency and safety of varying the frequency of whole blood donation (INTERVAL) study	8455 (48.8%)	44	European descent	Authors excluded related individuals in analyses	Multiple SNPs were significantly associated with circulating choline, choline phosphate, GPCs, PC, and SMs in the multi-cohort meta-analysis.
			European Prospective Investigation into Cancer and Nutrition(EPIC) -Norfolk study	11,539 (53.3%) total= 5841 (discovery) plus 5698 (validation)	59.8	European descent	Authors excluded related individuals in analyses	
Tabassum, 2019 [48]	Total lysoPC, lysoPC (14:0;0), lysoPC (16:1;0), PC (16:0;0–16:0;0), PC (16:0;0–16:1;0), PC (16:0;0–18:1;0), PC-ether (16:1;0–16:0;0), PC-ether 18:2;0–18:1;0, SM (32:1;2), SM (34:0;2), SM (38:2;2)	Genetic variants associated with 141 lipid species	The European Multicenter Study on Familial Dyslipidemias in Patients with Premature Coronary Heart Disease (EUFAM)	1039 (NR)	NR	Finnish	Families	Intronic variant rs151223356 near ROCK1 was significantly associated with circulating short acyl-chain lysoPC (14:0,0). Variants at DDX43, COL26A1, SYT1, and MAF were associated with circulating LysoPCs. SNPs near BLK, COL5A1, TNFAIP3, GATM, and RBFOX3 were significantly associated with circulating PCs. New associations of SNPs near GLTPD2, SYNE2, and CERS4 with circulating SM (34:0;2) were discovered.
			The Finnish National Study (FINRISK)	1142 (NR)	NR	Finnish	None	
Tahir, 2022 [49]	Betaine, predicted LysoPC, SMs	To identify novel locus-metabolite associations in Black individuals.	Jackson Heart Study(JHS)	2466 (62)	56	African American	Families	A SNP in the *SLC6A12* gene was significantly associated with circulating betaine. Rs334 (associated with the sickle mutation) was significantly associated with a circulating metabolite predicted to be LysoPC. Multiple SNPs were significantly associated with multiple circulating SMs.
Tukiainen, 2012 [50]	Choline, PC, SM	A set of 440,807 genotyped and imputed genetic markers are associated with 216 metabolic variables.	Northern Finland Birth Cohort 1966	4703 (31)	51	Finnish	None	No SNPs were significantly associated with choline/choline metabolites.
			The Cardiovascular Risk in Young Finns Study (YFS)	1904 (37.7)	54	Finnish	None	
			Helsinki Birth Cohort Study	708 (61.3)	60	Finnish	None	
			Genetics of METabolic Syndrome (GenMets) subsample of Finnish Health2000 examination survey	572 (55.8)	57	Finnish	None	
			The Dietary, Lifestyle, and Genetic determinants of Obesity and Metabolic Syndrome (DILGOM)	443 (50.1)	56	Finnish	None	
Yet, 2016 [51]	1-arachidonoylglycerophosphocholine, glycerolphosphocholine, PCs, LysoPCs, SMs	SNPS associated with high-throughput metabolic profiles	Twins United Kingdom (TwinsUK)	1001 (NR)	NR	British	Twins	SNPs were significantly associated with circulating LysoPC a C20:4 and 1-arachidonoyl-GPC across Biocrates and Metabolon platforms. Metabolite associations with genetic variants at the *SGPP1* locus did not match in name for PC aa C28:1 (Biocrates) and 1-stearoylglycerol (Metabolon).
Yousri, 2018 [52]	Choline, betaine, GPC, SMs	Loci affecting metabolites and metabolite ratios	Human subjects recruited from Hamad Medical Corporation (HMC) and HMC Primary Health Care Centers in Doha, Qatar	91 (45%)	50.1	Qatari	Authors accounted for relatedness in analyses	No SNPs were significantly associated with circulating choline or choline-related metabolites.
Yu, 2014 [53]	Betaine, GPCs, SMs	Common genetic variants influencing the metabolome in African Americans	The Atherosclerosis Risk in Communities (ARIC) Study	15,792 (65.2)	52.6	African American	None	No SNPs were significantly associated with any circulating choline-related metabolites.

CAD, coronary artery disease; CVD, cardiovascular disease; LysoPCs, lysophosphatidylcholine; PC, phosphatidylcholine; SM, sphingomyelin; T2D, type II diabetes; TMAO, trimethylamine N-oxide; BHMT, betaine-homocysteine S-methyltransferase; CBS, cystathionine beta synthase; CPS1, carbamoyl-phosphate synthase 1; GCKR, glucokinase regulator; LPA, lipoprotein(A); MYRF, myelin regulatory factor; NR, not reported; PDE4D; PROTE, EGCUT sub-cohort; ROCK1, rho associated coiled-coil containing protein kinase 1; SGPP1, sphingosine-1-phosphate phosphatase 1; SPTLC3, serine palmitoyltransferase long chain base subunit 3; SYNE2, spectrin repeat containing nuclear envelope protein 2; TMEM, transmembrane protein.

### MR studies

The 26 included MR studies utilized data from 157 total data sources representing 129 unique cohorts. Again, the majority of these cohorts included Europeans (70%), whereas the others represented transethnic populations (16%), Chinese (13%), and individuals from Australia in 1 cohort. The reported mean age of participants was 25 y or greater for all data sources, and the highest reported participant age was 72 y. Age was not reported for 47 data sources from 13 of the included studies. In the 157 reported data sources, the study population size ranged from 212 to 1,030,836 participants, where 49% of reported data sources had fewer than 5000 participants, 35% of data sources had 5000–100,000 participants, and the remaining 16% of data sources had >100,000 participants. Of the unique cohorts, 34% included unrelated individuals, 25% included related individuals (e.g., families, siblings, twins, or spouses), 6% included combination relatedness (used >1 cohort with different relatedness status), 10% of studies did not report relatedness, and for 25%, relatedness was not clear.

**FIGURE 2:**
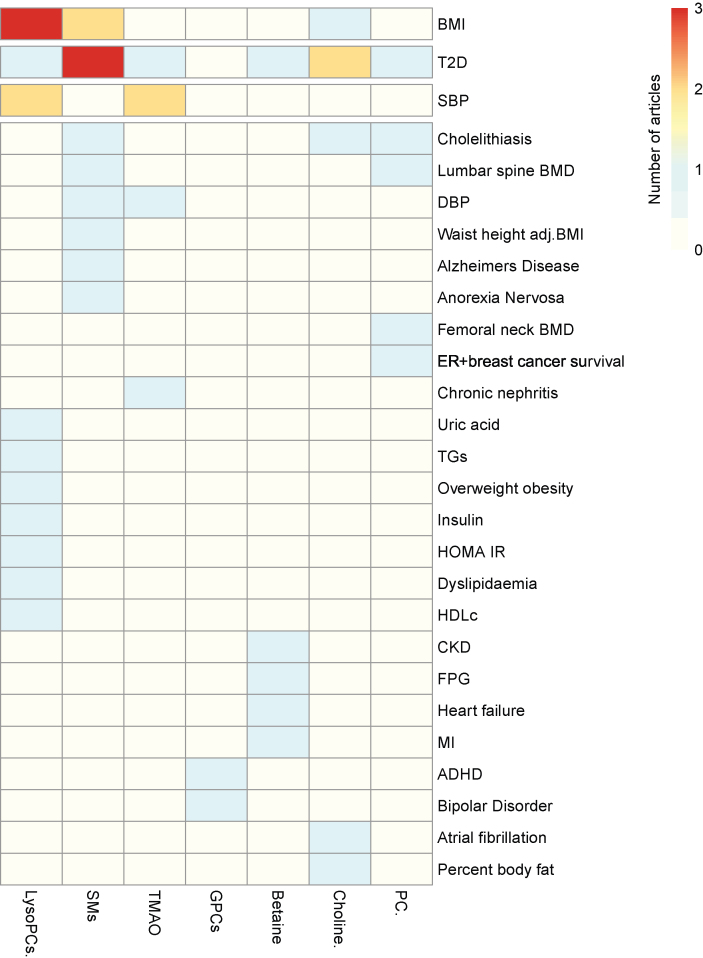
Heatmap of significant associations reported in Mendelian randomization studies. The scale and colors depict the number of articles reporting significant associations between a circulating choline-related metabolite and a health outcome. ^1^GPC, LysoPC, and SM include multiple metabolite forms: GPC (1-arachidonoyl-GPC, 1-docosahexaenoyl-GPC), LysoPC (total LysoPC, LysoPC 14:0/0:0, LysoPC 15:0, LysoPC 16:1), and SM (total SM, SM C34:0, SM C36:0, SM C34:1, SM (OH) C32:2, SM (2OH) C30:2). ADHD, attention-deficit/hyperactivity disorder; BMD, bone mass density; BMI, body mass index; CKD, chronic kidney disease; DBP, diastolic blood pressure; ER+, estrogen receptor positive; FPG, fasting plasma glucose; GPC, glycerophosphocholine; HDL cholesterol, high-density lipoprotein cholesterol; HOMA-IR, homeostasis model assessment of insulin resistance; LysoPC, lysophosphatidylcholine; MI, myocardial infarction; PC, phosphatidylcholine; SBP, systolic blood pressure; SM, sphingomyelin; T2D, type II diabetes; TG, triglyceride; TMAO, trimethylamine N-oxide.

MR studies investigated associations between 52 total unique choline metabolites and 66 unique health outcomes. Many included articles reported significant associations between multiple choline types and multiple health outcomes. Of these, 47 significant associations were reported between 16 metabolites (primarily choline, LysoPCs, PCs, betaine, and SMs) and 27 health outcomes. A complete summary of significant MR results is displayed in Table 3 [[18], [19], [54], [55], [60], [61], [64], [65], [66], [67], [68], [69], [71], [72], [73], [77]], Supplementary Table 2, and the Figure 2 heatmap. Important to the validity of genetically predicted exposures, a wide variety of ethnicities were investigated. A list of MR study characteristics and key outcomes is displayed in Table 4 [18, [54], [55], [56], [57], [58], [59], [60], [61], [62], [63], [64], [65], [66], [67], [68], [69], [70], [71], [72], [73], [74], [75], [76]]. Qualitative study characteristics, including cohort/data source, sample size, percentage of females, age, and ethnicity, are displayed in Supplementary Table 3.

**TABLE 3:** Choline/choline metabolites (*n* = 16) reported to have a significant association with a health outcome in Mendelian randomization studies

Choline/choline metabolite	Health outcomes
1- arachidonoyl-GPC	Bipolar disorder [61]
1-docosahexaenoyl-GPC	ADHD [71]
Betaine	T2D [60], fasting plasma glucose [66], heart failure, myocardial infarction, chronic kidney disease [64]
Choline	T2D, atrial fibrillation, percent body fat, and BMI [60] Cholelithiasis [65] T2D [19]
LysoPC	BMI [55]
LysoPC 14:0/0:0	BMI and pregnancy BMI [68]
LysoPC 15:0	Overweight/obesity, dyslipidemia, high uric acid, high insulin, high HOMA-IR, BMI, systolic blood pressure, uric acid, insulin, HOMA-IR, HDL cholesterol, and TG’s [69]
LysoPC 16:1	Pregnancy BMI [68]
PC	Cholelithiasis [65] T2D [19] Femoral neck BMD and lumbar spine BMD [77]
SM	ER+ breast cancer-specific survival [54] Waist-to-height adjusted BMI [55] Anorexia nervosa [61] Cholelithiasis [65] Alzheimer’s disease [67] Lumbar spine BMD [77]
SM C34:0	T2D [72]
SM C36:0	T2D [72]
SM C34:1	T2D [72]
SM (OH) C32:2	Systolic blood pressure, diastolic blood pressure, and BMI [73]
SM (2OH) C30:2	BMI [73]
TMAO	RR of elevated systolic blood pressure, RR chronic nephritis, RR T2D [64] Systolic blood pressure [18]

ADHD, Attention-deficit/hyperactivity disorder; BMI, body mass index; ER+, estrogen receptor positive; HDL cholesterol, high-density lipoprotein cholesterol; HOMA-IR, homeostasis model assessment of insulin resistance; LysoPC, lysophosphatidylcholine; PC, phosphatidylcholine; RR, relative risk; SM, sphingomyelin; T2D, type II diabetes; TG, triglyceride; TMAO, trimethylamine N-oxide; 1-arachidonoyl-GPC, 1-arachidonoyl-glycerophosphocholine; 1-docosahexaenoyl-GPC, 1-docosahexaenoyl-glycerophosphocholine.

**TABLE 4:** Individual study characteristics for included Mendelian randomization studies^1^

Author, y (reference)	MR type	Choline type(s) reported	Health outcome(s)	Key findings	Effect size for significant associations^2^
Adams, 2020 [54]	Two-sample MR	SM	Breast cancer-specific survival for estrogen receptor-negative (ER–) and ER+ survival	Circulating sphingomyelin was positively associated with ER+ breast cancer survival.	SM -> ER+ survival †: HR = 0.65; 95% CI: 0.45–0.94, *P* = 0.02 SM -> ER+ survival ††: HR = 0.65; 95% CI: 0.45–0.94, *P* = 0.02
Ahmad, 2022 [55]	Two-sample MR	PC, LysoPC, and SM	BMI and waist-to-height adjusted BMI (WHRadjBMI)	LysoPC was negatively associated with BMI. SM was negatively associated with WHRadjBMI.	LysoPC -> BMI † (combined PIVUS, ULSAM and TwinGene cohorts): β = −0.29; 95% CI: −0.52 to −0.050, *P* = 0.02 SM -> WHRadjBMI † (combined PIVUS, ULSAM and TwinGene cohorts): β = −0.51; 95% CI: −0.90 to −0.13, *P* = 8.00 × 10^−3^ SM -> WHRadjBMI † (combined ULSAM and PIVUS cohorts): β = −2.46; 95% CI: −3.78 to −1.14, *P* = 3.00 × 10^−4^ SM -> WHRadjBMI † (TwinGene cohort): β = −0.71; 95% CI: −1.3 to −0.12, *P* = 0.020
Del Greco, 2019 [56]	Two-sample MR	PC (38:3) and SMs	BMI and p-wave duration	No significant associations were reported for either health outcome. However, the association between circulating PC and BMI was near significant (β = 3.34, SE = 1.78, *P* = 0.057).	
Ganna, 2014 [57]	Two-sample MR	LysoPC (18:1), LysoPC (18:2), SM (28:1)	Coronary heart disease (CHD)	No significant associations were reported between circulating LysoPC 18:1, LysoPC 18:2, and SM 28:1 with CHD.	
Gong, 2021 [58]	One-sample MR	LysoPC	Total hip bone mass density (BMD) variation (femoral neck, trochanter, and intertrochanteric region)	No significant associations were reported between serum LysoPC and hip BMD variation.	
Guida, 2021 [59]	Two-sample MR	PC, LysoPC, SM, glycerophosphocholine (GPC)	Incident kidney cancer	No significant associations were reported between circulating PC, LysoPC, or GPC and incident kidney cancer.	
Jia, 2019 [60]	Bi-directional MR	TMAO, choline, betaine	Type II diabetes (T2D), atrial fibrillation, myocardial infarction, coronary artery disease (CAD), stroke, chronic kidney disease (CKD), BMI, % body fat, glycemic traits, lipids [HDL, LDL, total cholesterol, triglycerides (TGs)]	Plasma betaine was associated with T2D. Plasma choline was individually associated with T2D, atrial fibrillation, % body fat, and BMI.	Betaine -> T2D †: OR = 0.68; 95% CI: 0.48–0.95, *P* = 0.023 Choline -> T2D †: OR = 1.84; 95% CI: 1.00–3.42, *P* = 0.05 Choline -> % body fat †: β = –0.28, *P* = 0.013 Choline -> BMI ††: β = 0.33, *P* = 0.029 Choline -> atrial fibrillation +: OR = 1.57; 95% CI: 1.05–2.36, *P* = 0.028)
Jia, 2022 [61]	Two-stage MR (systematic MR and phenome-wide MR)	SM, 1-arachidonoyl-GPC	Anorexia nervosa, odds of bipolar disorder	Circulating SMs were associated with anorexia nervosa. Circulating 1-arachidonoyl-GPC was associated with the odds of bipolar disorder.	SM -> anorexia nervosa †: OR = 1.12; 95% CI: 1.06–1.19, *P* = 2.34 × 10^−5^ SM -> anorexia nervosa ++: OR = 1.13; 95% CI: 1.06–1.19, *P* = 4.37 × 10^−5^ SM -> anorexia nervosa +++: OR = 1.12; 95% CI: 1.07–1.19, *P* = 2.25 × 10^−5^ SM -> anorexia nervosa ++++: OR = 1.12; 95% CI: 1.06–1.19, *P* = 2.45 × 10^−5^ 1-arachidonoyl-GPC -> bipolar disorder †: OR = 0.31; 95% CI: 0.23–0.41, *P* = 3.52 × 10^−16^ 1-arachidonoyl-GPC -> bipolar disorder ++: OR = 0.31; 95% CI: 0.21–0.46, *P* = 6.76 × 10^−9^ 1-arachidonoyl-GPC -> bipolar disorder +++: OR = 0.30; 95% CI: 0.23–0.41, p = 9.55 × 10^−15^ 1-arachidonoyl-GPC -> bipolar disorder ++++: OR = 0.30; 95% CI: 0.23–0.41, *P* = 9.53 × 10^−15^
Liu, 2017 [62]	Two-sample bi-directional MR	PC alkyl-acyl C42:5, PC alkyl-acyl C44:4, PC alkyl-alkyl C44:5	Fasting blood glucose and T2D	No significant associations were reported between choline types and either health outcome (fasting blood glucose or T2D).	
Liu, 2022 [63]	Two-sample bi-directional MR	PC 32:1 and PC 40:5	Systolic blood pressure (SBP) and diastolic blood pressure (DBP)	No significant associations were reported between circulating PC 32:1 or PC 40:5 with SBP or DBP.	
Luo, 2022 [64]	Two-sample MR	Betaine, Choline, TMAO	Heart failure, T2D, atrial fibrillation, CAD, CKD, hypertension, valvular heart disease, valvular heart disease, myocarditis, myocardial infarction, hypertrophic cardiomyopathy, dilated cardiomyopathy	Circulating betaine was associated with relative risk of heart failure and myocardial infarction. For every 1 unit increase in betaine, the relative risk of heart failure and myocardial infarction increased, separately, yet relative risk of CKD decreased. For every 1 unit increase in circulating TMAO, relative risk of elevated SBP, chronic nephritis and T2DM increased. No significant associations were reported between choline and any health.	Betaine -> heart failure †: OR = 1.014; 95% CI: 1.002–1.026, *P* = 0.03 Betaine -> MI †: OR = 1.017; 95% CI: 1.001–1.033, *P* = 0.034 Betaine -> CKD †: OR = 0.963; 95% CI: 0.934–0.991, *P* = 0.01 TMAO -> blood pressure †: OR = 1.071; 95% CI: 1.010–1.136, *P* = 0.02 TMAO -> chronic nephritis †: OR = 1.031; 95% CI: 1.004–1.058, *P* = 0.024 TMAO -> T2D †: OR = 1.016; 95% CI: 1.002–1.033, *P* = 0.029
Mi, 2022 [65]	One-sample MR, two-sample MR, and bi-directional MR	SM, PC, choline	Cholelithiasis risk	Circulating PC, SM, and choline were all negatively associated with cholelithiasis risk.	Choline -> cholelithiasis risk (UK Biobank) †: β = –0.216 (SE: 0.101, *P* = 0.0331) Choline -> cholelithiasis risk (Finngen) †: β = –0.262 (SE: 0.111, *P* = 0.0186) SM -> cholelithiasis risk (UK Biobank) †: β = –0.213 (SE: 0.0856, *P* = 0.0128) SM -> cholelithiasis risk (Finngen) †: β = –0.223 (SE: 0.0846, *P* = 8.31 × 10^−3^ SM -> cholelithiasis risk (UK Biobank) ††: β = –0.342 (SE: 0.165, *P* = 0.0430) Choline -> cholelithiasis risk (UK Biobank) †††: β = –0.230 (SE: 0.0706, *P* = 0.00184) Choline -> cholelithiasis risk (Finngen) †††: β = –0.151 (SE: 0.0651, *P* = 0.0204) SM -> Cholelithiasis risk (UK Biobank) †††: β = −0.244 (SE: 0.0721, *P* = 7.04 × 10^−4^) SM -> cholelithiasis risk (Finngen) †††: β = –0.159 (SE: 0.0692, *P* = 0.0220) PC -> cholelithiasis risk (UK Biobank) †††: β = –0.209 (SE: 0.0660, *P* = 0.00152) PC -> cholelithiasis risk (Finngen) †††: β = –0.121 (SE: 0.0602, *P* = 0.0452)
Porcu, 2021 [66]	Two-sample bi-directional MR	Betaine, LysoPC	T2D	Circulating betaine was negatively associated with fasting plasma glucose. Circulating LysoPC was negatively associated with T2D.	Betaine -> fasting plasma glucose (method NR): β = –0.022 (SE: 0.005, *P* = 4.89 × 10^−5^) LysoPC -> T2D (method NR): β = –0.048 (SE: 0.012, *P* = 3.52 × 10^−5^)
Sun, 2022 [67]	Two-sample, two-stage MR	SM	Alzheimer’s disease (AD)	Each SD increase in circulating sphingomyelin was associated with a lower risk of Alzheimer’s disease.	SM -> Alzheimer’s disease †: OR = 2.53; 95% CI: 1.78–3.59, *P* = 2.10 × 10^−7^ SM -> Alzheimer’s disease ††: OR = 4.36; 95% CI: 2.39–7.94, *P* = 1.51 × 10^−6^ SM -> Alzheimer’s disease +++ : OR = 4.13; 95% CI: 3.91–4.37, *P* = 1.00 × 10^−299^ SM -> Alzheimer’s disease ++++ : OR = 2.78; 95% CI: 2.59–2.99, *P* = 7.44 × 10^−167^
Wang, 2022 (BMI and lipidomic biomarkers) [68]	One-sample MR	LysoPCs, Lysoalkyl-PCs, PCs SMs,	BMI and pregnancy BMI	Circulating LysoPC 14:0 was associated with BMI. Circulating LysoPC 14:0 and LysoPC 16:1 were associated with pregnancy BMI.	LysoPC 14:0 -> BMI ∗: SD difference = 0.20; 95% CI: 0.010–0.39, *P* = 0.042 LysoPC 14:0 -> pregnancy BMI ∗: SD difference = 0.37; 95% CI: 0.04–0.71, *P* = 0.029 LysoPC 16:1 -> Pregnancy BMI ∗: SD difference = 0.38; 95% CI: 0.020–0.74, *P* = 0.039
Wang, 2022 (TMAO and its precursors [18]	Two-sample MR	Betaine, choline, TMAO	SBP and DBP	A 1 unit increase in circulating TMAO was associated with a 1 SD increase in SBP, but not DBP. No significant associations were reported between betaine or choline with either SBP or DBP.	TMAO -> SBP †: β = 0.060 (SE: 0.029, *P* = 0.036) TMAO -> SBP ++++ : β = 0.062 (SE: 0.0270, *P* = 0.020) TMAO -> SBP ∗∗ : β = 0.060 (SE: 0.0290, *P* = 0.0420)
Wu, 2021 [69]	One-sample and two-sample MR	LysoPC 14:0, LysoPC 15:0	Metabolic syndrome, overweight/obesity,SBP, uric acid, insulin, fasting plasma glucose, HOMA-IR, BMI, TGs, HDL cholesterol, and Dyslipidemia	Higher concentrations of circulating LysoPC 15:0 was positively associated with the risk of overweight/obesity, dyslipidemia (particularly because of the increased serum TG concentrations), high uric acid, high insulin, and high HOMA-IR, but not with metabolic syndrome and high BP. No significant associations were reported between LysoPC 15:0 and BMI, SBP, or DBP in the 2-stage MR.	LysoPC 15:0 -> overweight/obesity † (one-sample): OR = 1.84; 95% CI: 1.10–3.08, *P* = 0.02 LysoPC 15:0 -> dyslipidaemia † (one-sample): OR = 1.78; 95% CI: 1.07–2.98, *P* = 0.0280 LysoPC 15:0 -> high uric acid † (one-sample): OR = 2.59; 95% CI: 1.36– 4.93, *P* = 4.00 × 10^−3^ LysoPC 15:0 -> high insulin † (one-sample): OR = 2.31; 95% CI: 1.27–4.18, *P* = 6.00 × 10^−3^ LysoPC 15:0 -> high HOMA-IR † (one-sample): OR = 2.28; 95% CI: 1.26–4.13, *P* = 7.00 × 10^−3^ LysoPC 15:0 -> BMI † (one-sample): β = 1.42; 95% CI: 0.480–2.35, *P* = 3.00 × 10^−3^ LysoPC 15:0 -> SBP † (one-sample): β = 5.72; 95% CI: 0.190–11.3, *P* = 0.0430 LysoPC 15:0 -> TGs † (one-sample): β = 0.540; 95% CI: 0.110–0.970, *P* = 0.0130 LysoPC 15:0 -> uric acid † (one-sample): β = 0.0600; 95% CI: 0.0600–0.0700, *P* = 0.0450 LysoPC 15:0 -> insulin † (one-sample): β = 1.52; 95% CI: 0.640–2.39, *P* = 1.00 × 10^−3^ LysoPC 15:0 -> HOMA-IR † (one-sample): β = 0.230; 95% CI: 0.0600–0.410, *P* = 1.00 × 10^−2^ LysoPC 15:0 -> HDL cholesterol † (two-sample): β = 0.147; 95% CI: 0.116–0.177, *P* = 1.00 × 10^−3^ LysoPC 15:0 -> TGs † (two-sample): β = 0.07; 95% CI: 0.05–0.10, p = 1.00 × 10^−3^
Xie, 2013 [70]	One-sample MR and two-sample MR	Betaine	Insulin sensitivity	No significant associations were reported between circulating betaine and insulin sensitivity (as measured by fasting insulin and T2D).	
Xu, 2022 [19]	Two-sample, 2-step MR	Choline, PC	CAD and T2D	Choline and PC were negatively associated with T2D. Choline and PC were not associated with CAD.	Choline -> T2D ∗∗: OR = 0.84; 95% CI: 0.76–0.93, *P* = 1.00 × 10^−3^ PC -> T2D †: OR = 0.87; 95% CI: 0.77–0.97, *P* = 0.016 PC -> T2D ∗∗: OR = 0.87; 95% CI: 0.80–0.94, *P* = 2.00 × 10^−3^
Yang, 2020 [71]	Two-sample MR	1-docosahexaenoyl-glyceropho-PC, betaine	Schizophrenia, major depression, bipolar disorder, autism spectrum disorder, attention-deficit/hyperactivity disorder(ADHD)	Circulating 1-docosahexaenoyl-glyceropho-PC was positively associated with ADHD in MR analysis. No significant associations were reported between 1-docosahexaenoyl-glyceropho-PC and schizophrenia, major depression, bipolar disorder, or autism spectrum disorder.	1-docosahexaenoyl-glyceropho-PC -> ADHD †: OR = 2.98; 95% CI: 1.75–5.07, *P* = 5.58 × 10^−5^
Yun, 2020 [72]	Two-sample MR	SM C34:0, SM C34:1, SM C36:0, SM C42:3	T2D	Circulating SMs C34:0, C36:0, and C34:1 were positively associated with T2D.	SM C34:0 -> T2D ++: OR = 0.86; 95% CI: 0.79–0.92, *P* = <0.001 SM C36:0 -> T2D ++: OR = 0.91; 95% CI: 0.85–0.98, *P* = 0.013 SM C34:1 -> T2D ++: OR = 1.19; 95% CI: 1.08–1.31, *P* = <0.001
Yun, 2022 [73]	Two-sample MR	PC, SMs	SBP and DBP	Circulating SM (OH) C32:2 was significantly associated with lower SBP and DBP, and a modest association with BMI. SM C32:1 was not associated with CAD.	SM (OH) C32:2 -> SBP +: β = –0.74; 95% CI: ^-^1.4 to −0.09, *P* = 0.026 SM (OH) C32:2 -> SBP ++: β = –0.57; 95% CI: ^-^0.85 to −0.28, p = 9.16 × 10^−5^ SM (OH) C32:2 -> SBP ∗∗∗ : β = –0.63; 95% CI: −1.16 to −0.10, *P* = 0.020) SM (OH) C32:2 -> SBP ∗∗∗∗ : β = –2.12; 95% CI: −4.06 to −0.18, *P* = 0.033 SM (OH) C32:2 -> DBP ++: β = –0.39; 95% CI: ^-^−0.59 to −0.20, *P* = 7.09 × 10^−5^ SM (OH) C32:2 -> DBP ∗∗∗ : β = –0.40; 95% CI: −0.74 to −0.05, *P* = 0.026 SM (OH) C32:2 -> BMI ++: β = –0.010; 95% CI: −0.03 to 0.00, *P* = 0.047) SM (OH) C32:2 -> BMI ∗∗∗∗ : β = –0.10; 95% CI: −0.19 to −0.020, *P* = 0.019 SM (2OH) C30:2 -> SBP ∗∗∗∗: β = –0.34; 95% CI: −0.63 to −0.050, *P* = 0.023)
Zhang, 2017 [74]	One-sample	LysoPC (18:2), LysoPC (18:1), SM	CAD	Circulating LysoPC (18:2), LysoPC (18:1), and SM were not associated with CAD risk.	
Zhang, 2021 [77]	Two-sample MR	LysoPC, PC, SM	Femoral neck BMD and lumbar spine BMD	Circulating PC was negatively associated with both femoral neck BMD and lumbar spine BMD. Circulating SM was negatively associated with lumbar spine BMD, but not femoral neck BMD.	PC -> Femoral neck BMD †: β = –0.066; 95% CI: NR, *P* = 6.98 × 10^−3^ PC -> Lumbar spine BMD †: β = –0.136; 95% CI: NR, *P* = 1.84 × 10^−7^ PC -> Lumbar spine BMD †††: β = −0.106; 95% CI: NR, *P* = 2.96 × 10^−3^ SM -> Lumbar spine BMD †: β= –0.145; 95% CI: NR, *P* = 0.0100 SM -> Lumbar spine BMD †††: β = −0.164; 95% CI: NR, *P* = 7.41 × 10^−3^
Zhuang, 2021 [75]	Two-sample bi-directional MR	Betaine, choline, TMAO	Alzheimer’s disease	Higher circulating TMAO, betaine, and choline were not significantly associated with the risk of AD/10 units after Bonferroni correction.	
Zhuang, 2022 [76]	Two-sample MR	Betaine, choline, TMAO	Inflammatory bowel disease, ulcerative colitis, and Crohn’s disease	Higher circulating betaine was reported as a near significant association with Crohn’s disease. Circulating choline and TMAO were not associated with any health outcome.	

BMI, body mass index; CI, confidence interval; HDL, high-density lipoprotein; HOMA-IR, homeostasis model assessment of insulin resistance; HR, hazard ratio; LysoPC, lysophosphatidylcholines; MR, Mendelian Randomization; OR, odds ratio; PC, phosphatidylcholines; SD, standard deviation; SE, standard error; SM, sphingomyelins; TMAO, trimethylamine N-oxide; 1-arachidonoyl-GPC, 1-arachidonoyl-glycerophosphocholine; 1-docosahexaenoyl-GPC, 1-docosahexaenoyl-glycerophosphocholine; AD, Alzheimer's disease; BP, blood pressure; MRPRESSO, Mendelian Randomization pleiotropy residual sum and outlier; MR-RAPS, Mendelian Randomization robust adjusted profile score; NR, not reported; PIVUS, Prospective Investigation of the Vasculature in Uppsala Seniors; ULSAM, Uppsala Longitudinal Study of Adult Men.

1 Only significant effect sizes were included.

2 The following symbols represent MR method: † inverse variance weighted; †† MR-Egger; ††† weighted median; †††† weighted mode; + mode-based estimate, ++ penalized-inverse variance weighted, +++ MR-RAPS, ++++ maximum likelihood, ∗ 2-stage least-squares regression, ∗∗ MRPRESSO, ∗∗∗ penalized weighted median, ∗∗∗∗ penalized robust MR-Egger

Regarding the current state of the literature, the following health outcome categories have been explored: cardiovascular, metabolic, brain and behavioral, kidney, bone, cancer, eye, lung, and gastrointestinal. Cardiovascular and metabolic health outcomes have been most frequently studied in relation to genetically predicted circulating choline/choline metabolites, with 9 cardiovascular [60,64,73] and 11 metabolic-related outcomes [18,19,55,60,66,68,69,73] reported to have significant associations. Metabolic-related outcomes include BMI, dyslipidemia, fasting plasma glucose, HDL cholesterol, HOMA-IR, insulin, overweight/obesity, percent body fat, type II diabetes (T2D), triglycerides, and waist-to-height adjusted BMI. Only 1 study explored the relationship with gastrointestinal health, and the results were not significant [76]. Four MR articles investigated genetically predicted choline/choline metabolites and kidney health outcomes, all reporting significant associations with various metabolites [60,64,65,69]. Two MR articles investigated bone health, and 1 study reported significant associations with PCs [58,77]. Lastly, 2 MR articles investigated cancer health outcomes, with 1 significant association between SM and estrogen receptor-positive breast cancer-specific survival [54,59].

## Discussion

The results of our scoping review detail numerous genetic loci associated with circulating choline and choline-related metabolites and their relationships to various health outcomes. These findings represent a rich dataset to be explored both in further genetic epidemiological investigations as well as experimental approaches, including *1*) cell-based functional analysis to determine the relevance of hits to choline handling across subcellular compartments; *2*) concordance of identified loci with genomic screens under conditions of varied choline nutriture; *3*) targeted analysis of the choline metabolome and choline handling (via isotope tracing) in transgenic animal models with whole-body and/or tissue-specific loss of identified loci; *4*) targeted analysis of the choline metabolome in humans across varied genotypes and genosets of identified loci under different choline intake conditions. A recent publication demonstrated the utility of utilizing GWAS hits of circulating choline and performing functional analyses of such hits to identify novel choline transporters, validating the utility of this dataset [78].

Notably, although this current investigation highlights the utility of examining existing genetic variant-metabolite relationships for identifying novel routes of investigation, this evidence base remains in its infancy. The studies and associations identified in this literature base do not represent cause and effect; however, they do identify areas for further research. Below, we highlight several key findings and observations across existing GWAS and MR studies.

### GWAS results

GWAS included in this scoping review report many genetic polymorphisms that are significantly associated with circulating choline and choline-related metabolite concentrations, revealing loci that influence the homeostatic processes maintaining circulating concentrations. As expected, given the diversity of metabolites captured in our search, the candidate loci that emerged reflect the diversity of physiological processes that choline-related metabolites intersect with, as well as the different analytic strategies (e.g., adjusting for blood lipid level when assessing genetic determinants of circulating lipid-soluble choline metabolites). Some identified loci are unsurprising and represent relatively direct metabolically proximal genes (fatty acid desaturase 1 (FADS1) association with PCs in 3 studies [31,40,44]), whereas other variants have less obvious links to circulating metabolites and may reveal novel distally related metabolic networks (spectrin repeat containing envelope protein 2 (SYNE2) association with SMs in 3 studies [40,44,48]). The key findings from our search extend other work reporting on single nucleotide polymorphisms associated with dietary choline deficiency [16], reporting on several genetic variant associations with lower circulating choline concentrations [27,38,43,47]. An important consideration is that the path from GWAS to biology is not straightforward because many loci contribute to standing genetic variation. Furthermore, single nucleotide polymorphisms shown to exert effects on metabolic pathways typically only contribute a small amount to metabolic insufficiency, which is often difficult to distinguish from background variation [79]. Studies exploring genetic risk scores with choline-related polymorphisms could help determine combined genetic risk and reveal a greater proportion of the variation in circulating choline-related metabolites that can be explained by genetics.

### MR studies results

Many metabolic health outcomes related to circulating choline metabolites with significant associations have been investigated across multiple populations. Common relationships assessed included BMI (in kg/m^2^), waist-to-height adjusted BMI, blood lipid concentrations, glucose-insulin dynamics, body composition (e.g., percent body fat), and T2D. LysoPCs were most associated with metabolic health outcomes, with betaine, choline, PCs, SMs, and TMAO also showing significant associations. Across MR studies, significant associations were most frequently reported for T2D, with genetically associated circulating betaine, choline, LysoPCs, PCs, SMs, and TMAO being the most widely investigated IVs. The risk of T2D varied between different choline and choline-containing metabolite concentrations, even within the same class of metabolites. For example, T2D risk was greater with higher genetically predicted serum choline [60], TMAO [64], and SM C34:1 [72]. However, in other studies, T2D risk was lower given higher genetically predicted serum choline and PC (mediated by sodium-glucose cotransporter 2 (SGLT2) inhibition), LysoPC, and SM C34:0 and C36:0 [19]. This phenomenon may highlight how the MR technique relies on all the assumptions necessary for unbiased IV analysis, including the requirement that there be no path from genotype to outcome except through the exposure of interest (i.e., the genotype can neither be confounded nor can it induce the outcome via other pathways [20]). The largest number of significant associations reported for a cardiovascular disease outcome was systolic blood pressure (SBP), showing relationships with LysoPCs [69], TMAO [18,64], SM (OH) C32:2[73], and SM (2OH) C30:2 [73]. Additionally, the direction of the association with a health outcome varied by metabolite. For example, genetically predicted TMAO [64] and LysoPCs [69] showed positive relationships with SBP, whereas SMs showed negative relationships with SBP [73]. As previously mentioned, 1 study investigating choline deficiency during pregnancy showed an increased risk of hypertension in rats [11]. However, the studies from this review did not identify an association between circulating choline concentrations and hypertension in adults. Although studies have shown that choline deficiency leads to liver dysfunction and muscle damage [6,11], no MR articles investigating these health outcomes were identified in this review.

Fewer articles investigating brain and behavioral outcomes were present in the peer-reviewed literature. Previous research has suggested a beneficial impact of higher dietary choline intake on brain/neurodevelopment [80,81], schizophrenia risk [10], and cognitive decline, as evidenced by differences in brain white matter hyperintensity volume [12]. We identified 4 brain and behavioral outcomes within MR studies that were shown to be associated with a genetically predicted circulating choline metabolite [Attention-deficit/hyperactivity disorder (glycerophosphocholine (GPC)) [71]; bipolar disorder (GPC) [61]; Alzheimer’s disease (SM) [67]; Anorexia Nervosa (SM) [61]]. In contrast, Alzheimer’s disease was not significantly associated with choline, betaine, or TMAO in Zhuang et al., 2021 [75], suggesting polymorphisms in SM production may be more critical to Alzheimer’s outcomes. Only 1 study reported on associations with genetically predicted GPC/betaine and major depression, autism spectrum disorder, and schizophrenia health outcomes, and associations were nonsignificant [71]. Considering the role of choline in sphingomyelin production and brain health, these outcomes may warrant further investigation.

### Strengths and limitations

This scoping review is limited in several ways. Individual genetic variants can have varying influences on health outcomes, having main, partial, or no effect at all. The existing GWAS literature examining genetic determinants of circulating metabolites remains in its infancy and is limited by predominantly semi-quantitative assessments of choline and its related metabolites, as well as limited capacity to identify specific molecular species (especially among the diverse lipid metabolites varying in their fatty acid composition). Improvement in identification and quantitation from untargeted approaches, as well as funding and interest for targeted analyses in large cohorts, will likely address these concerns in the future. Generally, across the GWAS-metabolomic literature, there is a great need to standardize approaches regarding sample matrices and collection strategies, quantitation of metabolites, statistical approaches (including covariate adjustment), and attempts to incorporate nutrient intake and nutritional status indicators into analyses. Indeed, the metabolome is strongly influenced by dietary intakes -- genetic variants can influence diet intakes, and dietary intakes can reveal or obscure metabolite-disease relationships, such as in the case of the methylenetetrahydrofolate reductase (MTHFR) C477T variant, its interaction with dietary folate/folic acid intake, and the risk of elevated homocysteine and cardiovascular disease.

With respect to MR analyses, it is likely that several of the existing genetic variants associated with choline metabolites are subject to the well-recognized limitations of MR, including weak instrument bias, pleiotropy, and confounding [20]. Confounding is of particular concern in the case of choline and choline-related metabolites, as circulating concentrations, especially unfasted samples, are sensitive to intake, and food sources of choline and its related metabolites are found across several food groups with intakes of additional nutrients/bioactive compounds that may influence disease relationships (e.g., animal-source foods for choline; grains and leafy greens for betaine; fish for TMAO). Only 1 MR study identified in this review explored dietary intake (lipidomic signatures of dairy consumption) in relation to a health outcome (blood pressure and cardiovascular disease risk) [73]. Of note, the majority of the studies included combined cohorts with both men and pre- and postmenopausal women. Estrogen modifies the phosphatidylethanolamine-*N*-methyltransferase enzyme, creating differences in dietary choline requirements for men and postmenopausal women [82]. Although included GWAS analyses adjusted for sex, the majority of MR analyses did not. Separating premenopausal women from men/postmenopausal women would likely identify sex-specific associations. In future analyses, participants should be analyzed according to sex and pre/postmenopausal status (separating premenopausal women from men/postmenopausal women), and hormone replacement therapy should be considered.

Lastly, our search strategy has a few limitations. Abstract and full-text screening may not have identified all relevant articles in which choline/choline metabolites are mentioned exclusively in supplemental files. Metabolome-wide analyses (particularly GWAS) have become more common, examining hundreds of circulating metabolite genome-wide associations. Authors report a small fraction of these associations within the main text, and publishing the full list of single nucleotide polymorphisms-x-metabolite associations would require the creation of a database. Lastly, ethnicity was largely European, which limits translation to other ethnicities.

In conclusion, there is a growing body of evidence illustrating the association of genetic polymorphisms with circulating choline and choline-related metabolite concentrations, as well as with numerous health outcomes. It is crucial to account for sex, menopausal status, and hormone replacement therapy in future analyses, specifically by separating premenopausal women and men/postmenopausal women. Future large GWAS and MR studies are needed that incorporate choline-related dietary intake into these types of analyses, as it may help to disentangle the varying consequences of genetic variants on circulating choline/choline metabolite concentrations and health outcomes.

## Author contributions

The authors’ responsibilities were as follows – TCW and MC: conceptualization; LEL, KCC, TCW, and MC: methodology; LEL and KCC: analyses with oversight from MC and TCW; LEL and KCC: data curation with oversight from TCW and MC; LEL, KCC, TCW, and MC: writing—original draft preparation; LEL, KCC, TCW, and MC: writing—review and editing; TCW: funding, and all authors: read and approved the final manuscript.

## Conflict of interest

Co-author KCK has received past research support for research related to choline from Blachem, Inc. and is an Associate Editor at the American Journal of Clinical Nutrition. Co-author TCW has received research support from the Egg Nutrition Center, Balchem, Inc., and National Cattlemen's Beef Association for his research on choline. All of his current and prior conflicts of interest can be found on his website www.drtaylorwallace.com.

## Funding

This work was supported by an Investigator-initiated unrestricted educational grant from the Egg Nutrition Center to Think Healthy Group, LLC. The sponsor had no role in the study design, the collection, analysis, and interpretation of data, the writing of the manuscript, or the decision of where to submit the article for publication. The authors and sponsor strictly adhere to the American Society for Nutrition’s guiding principles for private funding of food science and nutrition research.

## Data availability

All data are available in the manuscript and the supplementary materials.

## Appendix A Supplementary data

Multimedia component1 Multimedia component1
