# Supplementary material for: The Relationship of Circulating Choline and Choline-Related Metabolite Levels with Health Outcomes: A Scoping Review of Genome-Wide Association Studies and Mendelian Randomization Studies
Source: Adv Nutr. 2023 Dec 20;15(2):100164. doi: 10.1016/j.advnut.2023.100164 (PMC10819410; doi:10.1016/j.advnut.2023.100164)
Supplement: Multimedia component1 [file mmc1.docx]

The Relationship of Circulating Choline and Choline-Related Metabolite Levels with Health Outcomes: A Scoping Review of Genome-Wide Association Studies and Mendelian Randomization Studies

Lauren E. Louck, et al.

**Supplementary data**

**Search Strategy**

Database: Ovid MEDLINE(R) and Epub Ahead of Print, In-Process, In-Data-Review & Other Non-indexed Citations, Daily and Versions(R) <1946 to December 16, 2021>

Search Strategy:
--------------------------------------------------------------------------------
1 exp choline/ or choline.mp.
2 glycerophosphocholine.mp.
3 phosphocholine.mp.
4 exp Phosphatidylcholines/ or Phosphatidylcholines.mp.
5 phosphatidylcholine.mp.
6 exp Sphingomyelins/ or Sphingomyelins.mp.
7 sphingomyelin.mp.
8 choline bitartrate.mp.
9 exp Lecithins/ or Lecithins.mp.
10 lecithin.mp.
11 glycerolphosphocholine.mp.
12 or/1-11
13 exp Mendelian Randomization Analysis/ or Mendelian Randomization Analysis.mp.
14 Mendelian Randomization.mp.
15 Genetic instrumental variable analysis.mp.
16 or/13-15
17 12 and 16
***************************

**Table S1**. Publications excluded from the scoping review with exclusion reasons.

| **Author** | **Year** | **Title** | **Exclusion reason** |
| --- | --- | --- | --- |
| Abraham | 2009 | GWAS of late-onset Alzheimer's disease | Wrong publication type |
| Balkhiyarova | 2022 | 50th European Mathematical Genetics Meeting (EMGM) 2022 | Wrong publication type |
| Bernard | 2018 | Long-chain polyunsaturated fatty acids, gestation duration, and birth size: a Mendelian randomization study using fatty acid desaturase variants | Wrong study design |
| Bodhini and Mohan | 2018 | Mediators of insulin resistance & cardiometabolic risk: Newer insights | Wrong publication type |
| Burkhardt | 2015 | Integration of Genome-Wide SNP Data and Gene Expression Profiles Reveals Six Novel Loci and Regulatory Mechanisms for Amino Acids and Acylcarnitines in Whole Blood | No choline |
| Burri | 2010 | Putative gene loci for female sexual dysfunction in females: Results of a genome-wide association study in an unselected population | Wrong publication type |
| Cornelis | 2015 | Genome-wide association study of selenium concentrations | No choline |
| Curran | 2017 | Abstracts for the 41st Human Genetics Society of Australasia Annual Scientific Meeting Brisbane, Queensland August 5-8, 2017 | Wrong publication type |
| Davis | 2017 | Common, low-frequency, and rare genetic variants associated with lipoprotein subclasses and triglyceride measures in Finnish men from the METSIM study | No choline |
| de Oliveira Otto | 2018 | Genome-wide association meta-analysis of circulating odd-numbered chain saturated fatty acids: Results from the CHARGE Consortium | Wrong study design |
| Demirkan | 2012 | Genome-wide association study identifies novel loci associated with circulating phospho- and sphingolipid concentrations | Wrong study design |
| Dong | 2022 | CSF metabolites associated with biomarkers of Alzheimer’s disease pathology | Wrong publication type |
| Drouin-Chartier | 2022 | Plasma Lipidomic Profiles of Dairy Consumption: A New Window on Their Cardiometabolic Effects | Wrong publication type |
| Emmert | 2021 | Genetic and metabolic determinants of atrial fibrillation in a general population sample: The CHRIS Study | No choline |
| Freedman | 2021 | Prenatal prevention of psychiatric illness and childhood development population-wide | Wrong publication type |
| Ganna | 2013 | 1-Acyl-sn-glycero-3-phosphocholine levels are related to obesity and several markers of subclinical cv disease and its biosynthesis is associated with genetic variants in the 9p21 region | Wrong publication type |
| Grant | 2022 | Multi-Omics Characterization of Early- and Adult-Onset Major Depressive Disorder | No choline |
| Haase | 2015 | HDL Cholesterol and Risk of Type 2 Diabetes: A Mendelian Randomization Study | No choline |
| Hartiala | 2018 | GWAS of Plasma betaine levels | Wrong publication type |
| Hartiala | 2021 | Genome-wide analysis identifies novel susceptibility loci for myocardial infarction | No health outcome |
| Hicks | 2010 | GWAS of circulating sphingolipid concentrations in European populations | Wrong publication type |
| Hishida | 2021 | Genome-wide association study of serum prostate-specific antigen levels based on 1000 Genomes imputed data in Japanese: the Japan Multi-Institutional Collaborative Cohort Study | No choline |
| Hodgman | 2022 | Coenzyme A Restriction as a Factor Underlying Pre-Eclampsia with Polycystic Ovary Syndrome as a Risk Factor | Wrong publication type |
| Hong | 2013 | A genome-wide assessment of variability in human serum metabolism | Wrong study design |
| Hoxhaj | 2020 | The metabolic profile associated with head and neck cancer genetic variation and dietary intake | Wrong publication type |
| Huno | 2008 | Detecting new genes for tobacco-related cancers – genome-wide association study of lung cancer | Wrong publication type |
| Inouye | 2012 | Novel Loci for metabolic networks and multi-tissue expression studies reveal genes for atherosclerosis | Wrong study design |
| Khandaker | 2019 | Early-Life Biomarkers for Psychosis Risk in Young People: Another Nail in the Coffin for Cartesian Dualism | Wrong publication type |
| Khor | 2016 | Genome-wide association study identifies five new susceptibility loci for primary angle closure glaucoma | No choline |
| Kim | 2021 | Genome-Wide Gene-by-Smoking Interaction Study of Chronic Obstructive Pulmonary Disease | No choline |
| Kraus | 2015 | Metabolomic Quantitative Trait Loci (mQTL) Mapping Implicates the Ubiquitin Proteasome System in Cardiovascular Disease Pathogenesis. | No choline |
| Krumsiek | 2012 | Mining the unknown: a systems approach to metabolite identification combining genetic and metabolic information | Wrong study design |
| Kyriakou | 2021 | Common Genetic Aberrations Associated with Metabolic Interferences in Human Type-2 Diabetes and Acute Myeloid Leukemia: A Bioinformatics Approach | Wrong study design |
| Leon-Mimila | 2021 | Genome-Wide Association Study Identifies a Functional SIDT2 Variant Associated With HDL-C (High-Density Lipoprotein Cholesterol) Levels and Premature Coronary Artery Disease | No choline |
| Liu and Kiryluk | 2018 | Insights into CKD from Metabolite GWAS | Wrong publication type |
| Malaspina | 2020 | Hippocampal metabolite concentrations in schizophrenia vary in association with rare gene variants in the TRIO gene | Wrong publication type |
| Mittelstrass | 2011 | Discovery of sexual dimorphisms in metabolic and genetic biomarkers | No choline |
| Miyagawa | 2008 | Variant between CPT1B and CHKB associated with susceptibility to narcolepsy | No choline |
| Miyagawa | 2009 | Genome-wide association study of narcolepsy identifies a susceptibility variant located between CPT1b and CHKB | Wrong publication type |
| Nicholson | 2011 | A genome-wide metabolic qtl analysis in Europeans implicates two loci shaped by recent positive selection | Wrong study design |
| Ocampo-Medina | 2017 | 11th Congress of the International Society of Nutrigenetics/Nutrigenomics (ISNN): Abstracts | Wrong publication type |
| O'Sullivan | 2013 | Integration of metabolomic and genomic data highlights novel pathways for mechanistic exploration in coronary artery disease | Wrong publication type |
| Real | 2020 | ATP10B and the risk for Parkinson's disease | No choline |
| Ried | 2013 | Integrative genetic and metabolite profiling analysis suggests altered phosphatidylcholine metabolism in asthma | Wrong study design |
| Ried | 2014 | Novel genetic associations with serum level metabolites identified by phenotype set enrichment analyses | Wrong study design |
| Shim | 2015 | Pathway analysis based on a genome-wide association study of polycystic ovary syndrome | Wrong study design |
| Sonmez Flitman | 2019 | Integrating gene expression and metabolome data identifies genes causally acting on metabolite concentrations | Wrong publication type |
| Suhre | 2011 | Human metabolic individuality in biomedical and pharmaceutical research | No choline |
| Suvitaival | 2021 | Circulating sphingomyelin: A causal risk-factor of chronic kidney disease? | Wrong publication type |
| Toledo | 2016 | Genetic influence on levels of targeted metabolites associated with Alzheimer's disease | Wrong publication type |
| Wang | 2021 | Genome-wide association study of metabolites in patients with coronary artery disease identified novel metabolite quantitative trait loci. | Wrong publication type |
| Westerterp | 2016 | High-density lipoproteins, endothelial function, and mendelian randomization | Wrong publication type |
| Weston | 2022 | Investigating Genetic Determinants of Plasma Inositol Status in Adult Humans | Wrong study design |
| Xicoy | 2021 | Shared genetic etiology between Parkinson's disease and blood levels of specific lipids | Wrong study design |
| Xu | 2015 | A genome-wide systems analysis reveals strong link between colorectal cancer and trimethylamine N-oxide (TMAO), a gut microbial metabolite of dietary meat and fat | Wrong study design |
| Yao | 2019 | Evaluation of the causal association between blood metabolites and rheumatoid arthritis: A mendelian randomization study | Wrong publication type |
| Yazdani | 2019 | Genome analysis and pleiotropy assessment using causal networks with loss of function mutation and metabolomics | Wrong study design |
| Yin | 2022 | Genome-wide association studies of metabolites in Finnish men identify disease-relevant loci | Wrong study design |
| Yu | 2016 | Whole genome sequence analysis of serum amino acid levels. | No choline |
| Yu | 2016 | Loss-of-function variants influence the human serum metabolome | Wrong study design |
| Zhang | 2019 | Evaluation of the causal association between blood metabolites and osteoarthritis: A mendelian randomization study | Wrong publication type |
| Zhang | 2021 | Untargeted Metabolomics Study and Bidirectional Mendelian Randomization Analyses Identified Novel Metabolites Mediating the Effect of Current Smoking on Body Mass Index | Wrong publication type |

Table S2. Summary of total reported significant associations between choline/choline metabolites^1^ and a health outcome in MR studies.

| **Health outcomes** | **GPC** | **Betaine** | **Choline** | **LysoPC** | **PC** | **SM** | **TMAO** | **Row totals** |
| --- | --- | --- | --- | --- | --- | --- | --- | --- |
| Brain & behavioral health |  |  |  |  |  |  |  | 4 |
| *ADHD* | 1 |  |  |  |  |  |  | 1 |
| *Alzheimer’s disease* |  |  |  |  |  | 1 |  | 1 |
| *Anorexia Nervosa* |  |  |  |  |  | 1 |  | 1 |
| *Bipolar disorder* | 1 |  |  |  |  |  |  | 1 |
| Cardiovascular health |  |  |  |  |  |  |  | 9 |
| *Atrial Fibrillation* |  |  | 1 |  |  |  |  | 1 |
| *Diastolic blood pressure* |  |  |  |  |  | 1 | 1 | 2 |
| *Heart failure* |  | 1 |  |  |  |  |  | 1 |
| *Myocardial infarction* |  | 1 |  |  |  |  |  | 1 |
| *Systolic blood pressure* |  |  |  | 2 |  |  | 2 | 4 |
| Metabolic health |  |  |  |  |  |  |  | 24 |
| *BMI* |  |  | 1 | 3 |  | 2 |  | 6 |
| *Dyslipidaemia* |  |  |  | 1 |  |  |  | 1 |
| *Fasting plasma glucose* |  | 1 |  |  |  |  |  | 1 |
| *HDL-c* |  |  |  | 1 |  |  |  | 1 |
| *HOMA-IR* |  |  |  | 1 |  |  |  | 1 |
| *Insulin* |  |  |  | 1 |  |  |  | 1 |
| *Overweight/obesity* |  |  |  | 1 |  |  |  | 1 |
| *Percent body fat* |  |  | 1 |  |  |  |  | 1 |
| *T2D* |  | 1 | 2 | 1 | 1 | 3 | 1 | 9 |
| *Triglycerides* |  |  |  | 1 |  |  |  | 1 |
| *Waist-to-height adjusted BMI* |  |  |  |  |  | 1 |  | 1 |
| Kidney health |  |  |  |  |  |  |  | 6 |
| *Cholelithiasis* |  |  | 1 |  | 1 | 1 |  | 3 |
| *Chronic kidney disease* |  | 1 |  |  |  |  |  | 1 |
| *Chronic nephritis* |  |  |  |  |  |  | 1 | 1 |
| *Uric acid* |  |  |  | 1 |  |  |  | 1 |
| Bone health |  |  |  |  |  |  |  | 3 |
| *Femoral neck BMD* |  |  |  |  | 1 |  |  | 1 |
| *Lumbar spine BMD* |  |  |  |  | 1 | 1 |  | 2 |
| Cancer |  |  |  |  |  |  |  | 1 |
| *ER+ breast cancer-specific survival* |  |  |  |  |  | 1 |  | 1 |
| **Column totals** | 2 | 5 | 6 | 13 | 4 | 12 | 5 |  |

GPC=glycerophosphocholine, LysoPC=lysophosphatidylcholine, PC=phosphatidylcholine, SM=sphingomyelin, T2D=type II diabetes, TMAO=trimethylamine N-oxide

^1^GPC, LysoPC, and SM include multiple metabolite forms: GPC (1-arachidonoyl-GPC, 1-docosahexaenoyl-GPC), LysoPC (total LysoPC, LysoPC 14:0/0:0, LysoPC 15:0, LysoPC 16:1), and SM (total SM, SM C34:0, SM C36:0, SM C34:1, SM (OH) C32:2, SM (2OH) C30:2).

Table S3. Population characteristics for included MR studies.

| **Author, year (ref)** | **Cohort or data source** | **Sample size (% female)** | **Mean age or range**, **y** | **Ethnicity** |
| --- | --- | --- | --- | --- |
| Adams, 2020 ([1](#_ENREF_1)) | Kettunen et al. (2016) GWAS data | 13,476 (NR) | NR | Finnish, Estonian, Dutch, German |
|  | Guo et al. (2015) breast cancer-specific survival GWAS data | ~29940 (NR) | NR | American, British, Finnish, Swedish, Netherlands, German, Irish, Danish, Australian, Belgian, Canadian, European, International |
| Ahmad, 2022 ([2](#_ENREF_2)) | Uppsala Longitudinal Study of Adult Men (ULSAM) | 1,135 (0%) | 70.9 | Swedish |
|  | Prospective Investigation of the Vasculature in Uppsala Seniors (PIVUS) | 970 (50%) | 70.2 | Swedish |
|  | TwinGenes cohort | 2,059 (42%) | 68.6 | Swedish |
|  | Cooperative Health Research in the Region Augsburg (KORA) and Twins United Kingdom (TwinsUK) metabolites GWAS by Shin et. al., 2014 | 7,824 (~ 83.5%) | ~55.1 | German, British |
|  | Cohorts for Heart and Aging Research in Genomic Epidemiology (CHARGE) Consortium GWAS by Guan et. al., 2014 | 8,631 (55%) | 45.8-72.0 | European |
|  | The Framingham Heart Study (FHS) metabolites GWAS by Rhee et. al., 2013 | 2,076 (51%) | 45.0-65.0 | European |
| Del Greco, 2019 ([3](#_ENREF_3)) | Orkney Complex Disease Study (ORCADES) | 951 (56%) | 53 | French |
|  | Microisolates in South Tyrol Study (MICROS) | 839 (52%) | 44 | Italian |
|  | European Special Population Research Network (EUROSPAN) GWAS of %PC 38:3 concentrations by Del Greco et. al., 2012 | 4034 (NR) | NR | Swedish, Scottish, Croatian, Dutch, Italian, German, Netherlands, Italian, |
|  | Genetic Investigation of Anthropometric Traits (GIANT) Consortium  + UKBiobank GWAS by Yengo et. al., 2018 | 734,481 (NR) | NR | European |
| Ganna, 2014 ([4](#_ENREF_4)) | TwinGenes cohort | 1,670 (52%) | 59.2-69.9 | Swedish |
|  | Prospective Investigation of the Vasculature in Uppsala Seniors (PIVUS) | 970 (50%) | 70.1-71.0 | Swedish |
|  | Uppsala Longitudinal Study of Adult Men (ULSAM) | 1,028 (0%) | 70.1-71.0 | Swedish |
| Gong, 2021 ([5](#_ENREF_5)) | Peri- and postmenopausal Chinese women who were enrolled from local communities from June 2014 to January 2018 | 517 (100%) | 52.85 | Chinese |
| Guida, 2021 ([6](#_ENREF_6)) | European Prospective Investigation into Cancer and Nutrition (EPIC) | 1,270 (44.4% [pooled across cohorts]) | 57.6 | European |
|  | The Melbourne Collaborative Cohort Study (MCCS) | 280 (44.4% [pooled across cohorts]) | 57.7 | Australian |
|  | Northern Sweden Health and Disease study (NSHDS) | 326 (44.4% [pooled across cohorts]) | 57.8 | Swedish |
|  | University of Tartu—Estonian Biobank (Estonian BB) | 230 (44.4% [pooled across cohorts]) | 57.9 | Estonian |
|  | The Trøndelag Health Study (HUNT) | 508 (44.4% [pooled across cohorts]) | 57.1 | Norwegian |
| Jia, 2019 ([7](#_ENREF_7)) | Diabetes Genetics Replication and Meta-analysis (DIAGRAM) Consortium | 149, 821 (NR) | NR | European |
|  | Atrial Fibrillation Consortium (AFGen) | 588190 (NR) | NR | Transethnic |
|  | Coronary Artery Disease Genomewide Replication and Meta-analysis (CARDIoGRAM) plus the Coronary Artery Disease (C4D) Genetics (CARDIoGRAMplusC4D) Consortium | 171873 (NR) | NR | Transethnic |
|  | Coronary Artery Disease Genomewide Replication and Meta-analysis (CARDIoGRAM) plus the Coronary Artery Disease (C4D) Genetics (CARDIoGRAMplusC4D) Consortium | 184305 (NR) | NR | Transethnic |
|  | National Institute of Neurological Disorders and Stroke Genetics Network (SiGN) and International Stroke Genetics Consortium (ISGC) | 435001 (NR) | NR | Transethnic |
|  | Chronic Kidney Disease Consortium (CKDGen) | 133,814 (NR) | NR | European |
|  | Genetic Investigation of Anthropometric Traits (GIANT) Consortium | 339,224 (NR) | NR | European |
|  | Meta-Analyses of Glucose and Insulin-Related Traits Consortium (MAGIC) | 133,010 (NR) | NR | European |
|  | Global Lipids Genetics Consortium (GLGC) | 188,578 (NR) | NR | Transethnic |
| Jia, 2022 ([8](#_ENREF_8)) | CHOP/PFCG; GCAN/WTCCC3; GCAN/WTCCC3/IARC/CNG; ANGI-DK; ANGI-ANZ/QSkin; ANGI-US/PFCG; ANGI-SE (Riksät/SCÄ/LifeGene/other); UK Biobank | 72,517 (NR) | NR | American/Canadian, British, German, Italian; Finnish, French, German, Norwegian, Swedish, Southern Italian, Greek, Dutch, Spanish, British, Amer-ican/Canadian; Polish, Czech; Danish; Australian, New Zealander; American; Swedish, Danish; British |
|  | BOMA-Australia; BOMA-Germany II; BOMA-Germany III; BOMA-Poland; BOMA-Spain; BOMA-Germany I; Trinity College Dublin; University of Edinburgh; FaST, TGEN1,2; French; BiGs: GAIN; BACCS; Nova Scotia, MGH i2b2 controls; ICCBD BDRN; Janssen, SAGE controls; Mayo Clinic; Pritzker Neuropsychiatric Disorders Research Consortium (U Michigan); Pfizer; BOMA-Romania; STEP2, MIGEN controls; STEP1; SWEBIC I Affy; SWEBIC I IllumiNR; TOP; TOP, additioNRl samples on new platform; Dutch-UCLA; University College London; UMEA; ICCBD USC; WTCCC; Bulgarian trios; UK trios; Janssen; PsyCourse; BIGs/TGEN; Bi-polar Depression Treatment Response Study; dutcha; gawlia; GUF-Bipolar; GPC; NeuRA-psych (including controls from The Australian Schizophrenia Research Bank); spsp3a; GEMS and EIMS; ukwa1a; usaw4a; usaw5a; iPSYCH; deCODE; Estonian Biobank; Nord-Trøndelag Health Study; UK Biobank; BOMA-Russia | 413,466 (NR) | NR | Australian; German; German; Polish; Spanish; German; British; British; American; French; American; American; Canadian/ American; British; American; American; American; American; Romanian; American; American; Swedish; Swedish; Norwegian; Norwegian; Dutch; British; Swedish; American; British; Bulgarian; British; East. European; German; American; German; Dutch; German; German; American; Australian; Spanish; Swedish; British; American; Canadian/ American; Danish; Icelander; Estonian; Norwegian; British; Russian; Greek; Austrian; Romanian |
| Liu, 2017 ([9](#_ENREF_9)) | Combined cases (Erasmus Rucphen Family (ERF) study metabolite GWAS, Meta-Analyses of Glucose and Insulin-related traits Consortium (MAGIC), DIAbetes Genetics Replication and Meta-Analysis (DIAGRAM) Consortium) | 212  (49.1%) | 59.8 | European |
|  | Combined controls (Erasmus Rucphen Family (ERF) study metabolite GWAS, Meta-Analyses of Glucose and Insulin-related traits Consortium (MAGIC), DIAbetes Genetics Replication and Meta-analysis (DIAGRAM) Consortium) | 2,564 (55.9%) | 48.2 | European |
| Liu, 2022 ([10](#_ENREF_10)) | Study conducted on the island of Vis, Croatia (CROATIA-Vis) | 710 (57.4%) | 56.6 | European |
|  | Erasmus Rucphen Family (ERF) | 717 (59.0%) | 51.9 | European |
|  | Northern Swedish Population Health Survey (NSPHS) | 678 (53.2%) | 47.1 | Swedish |
|  | Orkney Complex Disease Study (ORCADES) | 681 (57.9%) | 57.0 | European |
|  | Microisolates in South Tyrol Study (MICROS) | 1,185 (56.0%) | 45.7 | European |
| Luo, 2022 ([11](#_ENREF_11)) | Framingham Heart Study (FHS) | 2076  (51%) | 55 | European |
|  | Heart Failure Molecular Epidemiology for Therapeutic Targets Consortium GWAS meta-analysis by Shah et. al., 2020 | 977,323(NR) | NR | European |
|  | Diabetes Genetics Replication and Meta-analysis (DIAGRAM) Consortium for T2DM | 149,821 (NR) | NR | European |
|  | Atrial Fibrillation Consortium (AFGen) | 1,030,836  (NR) | NR | Norwegian, Icelandic, American, European, British |
|  | Coronary Artery Disease Genome-wide Replication and Meta-analysis (CARDIoGRAM) plus the Coronary Artery Disease (C4D) Genetics (CARDIoGRAMplusC4D) Consortium | 396,525 (NR) | NR | Transethnic |
|  | Chronic Kidney Disease Consortium (CKDGen) | 133,814 (NR) | NR | European |
|  | International Consortium of Blood Pressure-Genome Wide Association Studies (ICBP) | 299,024 (NR) | NR | Transethnic |
|  | GWAS for valvular heart disease | 178,726 (NR) | NR | Japanese, Finnish, British |
|  | GWAS for myocarditis | 177,847 (NR) | NR | Japanese, Finnish, British |
|  | GWAS for hypertrophic cardiomyopathy | 177,745 (NR) | NR | Transethnic |
|  | GWAS for dilated cardiomyopathy | 353,937 (NR) | NR | Transethnic |
| Mi, 2022 ([12](#_ENREF_12)) | Finnish Genetics of Intelligence (FinnGen) study (Round 5) | 214,167(NR) | NR | Finnish |
|  | UK Biobank | 300791 (NR) | NR | European |
|  | 123 circulating metabolite levels by Kettunen et al. 2016 | 25,000  (NR) | NR | Finnish, Estonian, Dutch, German |
| Porcu, 2021 ([13](#_ENREF_13)) | Caucasian population of Lausanne, Switzerland (CoLaus) study (baseline) | 788 (34.0%) | 56.2 | Swiss |
|  | Caucasian population of Lausanne, Switzerland (CoLaus) study (year 5) | 788 (34.0%) | 61.8 | Swiss |
|  | Caucasian population of Lausanne, Switzerland (CoLaus) study (year 10) | 673 (35.7%) | 66.7 | Swiss |
|  | French Cohort of Undifferentiated Spondyloarthritis (DESIR) (baseline) | 984 (47.7%) | 48.2 | European |
|  | French Cohort of Undifferentiated Spondyloarthritis (DESIR) (year 3) | 961 (47.3%) | 51.2 | European |
|  | French Cohort of Undifferentiated Spondyloarthritis (DESIR) (year 6) | 932 (47.4%) | 54.2 | European |
|  | French Cohort of Undifferentiated Spondyloarthritis (DESIR) (year 9) | 957 (48.3%) | 57.2 | European |
|  | GWAS performed for 453 whole blood metabolites by Shin et. al., 2014 | 7824 (NR) | 57.0 | European |
| Sun, 2022 ([14](#_ENREF_14)) | Human Blood Metabolites GWAS for IV by Shin et. al, 2014 | 7,824 (NR) | 57.0 | German, British |
|  | Human Blood Metabolites GWAS for IV by Kettunen et. al., 2016 | 24,925 (NR) | NR | Finnish, Estonian, Dutch, German |
|  | Human Blood Metabolites GWAS for IV by Borges et. Al., 2020 | 115,078 (NR) | NR | European |
|  | International Genomics of Alzheimer’s Project (IGAP) | 63,926 (NR) | NR | European |
| Wang, 2022 (BMI & Lipidomic Biomarkers) ([15](#_ENREF_15)) | Tongji-Shuangliu Birth Cohort | 1,008 (100%) | 27.8 | Chinese |
| Wang, 2022 (TMAO & Its Precursors ([16](#_ENREF_16)) | Framingham Heart Study (FHS) GWAS of human metabolome by Rhee et. al., 2013 | 2,076 (51%) | 55 | European |
|  | International Consortium of Blood Pressure Genome-Wide Association Studies were obtained from a GWAS meta-analysis | 757,601(NR) | NR | European |
| Wu, 2021 ([17](#_ENREF_17)) | Subset of cross-sectional survey on Metabolic Syndrome in Hangzhou, Zhejiang, China as the discovery cohort by Zhu et. al., 2017 | 240  (39.2%) | 62.4 | Chinese |
|  | Subset of cross-sectional survey on Metabolic Syndrome in Hangzhou, Zhejiang, China for internal validation cohort by Zhu et. al., 2017 | 1,157 (49.5%) | 56.8 | Chinese |
|  | Xiazhi sub-cohort: sub-population of the Zhejiang Metabolic Syndrome Cohort for external replication | 402 (59.5%) | 58.6 | Chinese |
| Xie, 2013 ([18](#_ENREF_18)) | Rapid Increase in Childhood Obesity (RISC) study | 957 (NR) | 43.91 | European |
|  | Botnia Study (GWAS replication) | 341 (NR) | 46.98 | European |
|  | European network on Functional Genomics of type 2 diabetes (EUGENE2) Consortium | 577  (NR) | NR | European |
|  | Stanford Insulin Suppression Test (IST) Cohort | 263  (NR) | NR | European |
| Xu, 2022 ([19](#_ENREF_19)) | UK Biobank 2018 (SLGT2 genetic variants) | 344,182 (NR) | NR | European |
|  | UK Biobank 2020 (choline metabolites) | 114,999 (NR) | NR | European |
|  | Finnish Genetics of Intelligence (FinnGen) study (choline metabolites) | ~6,135 (NR) | 45–74 | Finnish |
|  | Finnish Genetics of Intelligence (FinnGen) study (T2D) | 251,339 (NR) | NR | Finnish |
|  | Diabetes Genetics Replication and Meta-analysis (DIAGRAM) Consortium | 972,254 (NR) | NR | European |
|  | Coronary Artery Disease Genome-wide Replication and Meta-analysis (CARDIoGRAM) plus the Coronary Artery Disease (C4D) Genetics (CARDIoGRAMplusC4D) Consortium  with UK Biobank (Coronary Artery Disease [less than 70% MI; others including PTCA, CABG, IHD and angina, etc.]) | 332,477 (NR) | 40–69 | Transethnic |
|  | Coronary Artery Disease Genome-wide Replication and Meta-analysis (CARDIoGRAM) plus the Coronary Artery Disease (C4D) Genetics (CARDIoGRAMplusC4D) Consortium  (Coronary artery disease [~70%MI; 30% others including acute coronary syndrome, chronic stable angina, or coronary stenosis >50%]) | 184,305 (NR) | NR | Transethnic |
| Yang, 2020 ([20](#_ENREF_20)) | Metabolite GWAS by Shin et. al., 2014 | 7,824  (NR) | 57.0 | European |
|  | Psychiatric Genomics Consortium (PGC), schizophrenia (Consortium SGotPG, 2014) | 150,064 (NR) | NR | Transethnic |
|  | Howard et al. 2019 Nature Neuroscience, major depressive disorder | 807,553 (NR) | NR | Transethnic |
|  | Stahl et al. 2019 Nature Genetics, bipolar disorder | 51,710 (NR) | NR | European |
|  | Grove et al. 2019 Nature Genetics, autism spectrum disorder | 46,350  (NR) | NR | European |
|  | Martin et al. 2018 Biological Psychiatry, ADHD | Cases= 20,183 (25%); Controls= 35,191 (38%) | NR | Transethnic |
| Yun, 2020 ([21](#_ENREF_21)) | Nutrition and Health of Aging Population in China (NHAPC) study | 1,974 (~58.3%) | 50-70 | Chinese |
|  | China Health and Nutrition Survey | ~5,731 (NR) | NR | Chinese |
| Yun, 2022 ([22](#_ENREF_22)) | Nutrition and Health of Aging Population in China (NHAPC) study | 2,140 (~59.4%) | 50-70 | Chinese |
|  | Dietary Pattern and Metabolic Health (DPMH) study (replication cohort) | 212 (~14.2%) | 25-60 | Chinese |
| Zhang, 2017 ([23](#_ENREF_23)) | Category 1 cohort: longitudinal male cohort | 1,272 (0%) | 71.2 | Chinese |
|  | Category 2 cohort: community cohort in Shandong, China | 1,010 (50%) | 70.2 | Chinese |
|  | Category 3 cohort: longitudinal cohort study, twins born in Shandong, China | 1,810 (52%) | 65.11 | Chinese |
| Zhang, 2021 ([24](#_ENREF_24)) | Framingham Heart Study Offspring (FOS) | 1,152 (54.2%) | 55.4 | European |
|  | Hong Kong Osteoporosis Study (HKOS) | 634 (82.8%) | 52.1 | Chinese |
| Zhuang, 2021 ([25](#_ENREF_25)) | Genome-wide meta-analysis for clinically diagnosed AD by Jansen et al., 2019 | 455,258(~54.5%) | NR | European |
|  | GWAS of the human metabolome using Framingham Offspring Cohort (FOS) by Rhee et al., 2013 | 2076 (51%) | 55 | European |
| Zhuang, 2022 ([26](#_ENREF_26)) | Framingham Heart Study (FHS) | 2,076 (51%) | 55 | European |
|  | International IBD Genetics Consortium | 86,640 (NR) | NR | European |

This table contains population characteristics that correspond to **Table 4** from the main manuscript.

**Supplementary References**

1. Adams CD. Circulating sphingomyelins on estrogen receptor-positive and estrogen receptor-negative breast cancer-specific survival. Breast Cancer Management. 2020;9(3).

2. Ahmad S, Hammar U, Kennedy B, Salihovic S, Ganna A, Lind L, et al. Effect of General Adiposity and Central Body Fat Distribution on the Circulating Metabolome: A Multicohort Nontargeted Metabolomics Observational and Mendelian Randomization Study. Diabetes. 2022;71(2):329-39.

3. Del Greco FM, Foco L, Teumer A, Verweij N, Paglia G, Meraviglia V, et al. Lipidomics, Atrial Conduction, and Body Mass Index Evidence From Association, Mediation, and Mendelian Randomization Models. Circulation-Genomic and Precision Medicine. 2019;12(7).

4. Ganna A, Hedman AK, Magnusson PKE, Arnlov J, Fall T, Ingelsson E, et al. Large-scale Metabolomic Profiling Identifies Novel Biomarkers for Incident Coronary Heart Disease. Figshare. 2014.

5. Gong R, Xiao HM, Zhang YH, Zhao Q, Su KJ, Lin X, et al. Identification and Functional Characterization of Metabolites for Bone Mass in Peri-and Postmenopausal Chinese Women. Journal of Clinical Endocrinology and Metabolism. 2021;106(8):E3159-E77.

6. Guida F, Tan VY, Corbin LJ, Smith-Byrne K, Alcala K, Langenberg C, et al. The blood metabolome of incident kidney cancer: A case-control study nested within the MetKid consortium. Plos Medicine. 2021;18(9).

7. Jia J, Dou P, Gao M, Kong X, Li C, Liu Z, et al. Assessment of Causal Direction Between Gut Microbiota-Dependent Metabolites and Cardiometabolic Health: A Bidirectional Mendelian Randomization Analysis. Diabetes. 2019;68(9):1747-55.

8. Jia Y, Hui L, Sun L, Guo D, Shi M, Zhang K, et al. Association Between Human Blood Metabolome and the Risk of Psychiatric Disorders. Schizophrenia bulletin. 2022.

9. Liu J, Klinken JBV, Semiz S, Dijk KWV, Verhoeven A, Hankemeier T, et al. A Mendelian Randomization Study of Metabolite Profiles, Fasting Glucose, and Type 2 Diabetes. Diabetes. 2017;66(11):2915-26.

10. Liu J, de Vries PS, Del Greco FM, Johansson A, Schraut KE, Hayward C, et al. A multi-omics study of circulating phospholipid markers of blood pressure. Scientific Reports. 2022;12(1).

11. Luo Q, Hu Y, Chen X, Luo Y, Chen J, Wang H. Effects of Gut Microbiota and Metabolites on Heart Failure and Its Risk Factors: A Two-Sample Mendelian Randomization Study. Frontiers in Nutrition. 2022;9.

12. Mi J, Jiang L, Liu Z, Wu X, Zhao N, Wang Y, et al. Identification of blood metabolites linked to the risk of cholelithiasis: a comprehensive Mendelian randomization study. Hepatology International. 2022.

13. Porcu E, Gilardi F, Darrous L, Yengo L, Bararpour N, Gasser M, et al. Triangulating evidence from longitudinal and Mendelian randomization studies of metabolomic biomarkers for type 2 diabetes. Scientific Reports. 2021;11(1):6197.

14. Sun X, Zhang H, Shan M, Dong Y, Zhang L, Chen L, et al. Comprehensive Transcriptome Analysis of Patients With Keratoconus Highlights the Regulation of Immune Responses and Inflammatory Processes. Frontiers in Genetics. 2022;13.

15. Wang Y, Wu P, Huang Y, Ye Y, Yang X, Sun F, et al. BMI and lipidomic biomarkers with risk of gestational diabetes in pregnant women. Obesity. 2022.

16. Wang H, Luo Q, Ding X, Chen L, Zhang Z. Trimethylamine N-oxide and its precursors in relation to blood pressure: A mendelian randomization study. Frontiers in cardiovascular medicine. 2022;9:922441.

17. Wu Q, Li J, Sun X, He D, Cheng Z, Li J, et al. Multi-stage metabolomics and genetic analyses identified metabolite biomarkers of metabolic syndrome and their genetic determinants. EBioMedicine. 2021;74:103707.

18. Xie W, Wood AR, Lyssenko V, Weedon MN, Knowles JW, Alkayyali S, et al. Genetic Variants Associated With Glycine Metabolism and Their Role in Insulin Sensitivity and Type 2 Diabetes. Diabetes. 2013;62(6):2141-50.

19. Xu M, Zheng J, Hou T, Lin H, Wang T, Wang S, et al. SGLT2 Inhibition, Choline Metabolites, and Cardiometabolic Diseases: A Mediation Mendelian Randomization Study. Diabetes care. 2022.

20. Yang J, Yan B, Zhao B, Fan Y, He X, Yang L, et al. Assessing the causal effects of human serum metabolites on 5 major psychiatric disorders. Schizophrenia Bulletin. 2020;46(4):804-13.

21. Yun H, Sun L, Wu Q, Zong G, Qi Q, Li H, et al. Associations among circulating sphingolipids, beta-cell function, and risk of developing type 2 diabetes: A population-based cohort study in China. PLoS Medicine / Public Library of Science. 2020;17(12):e1003451.

22. Yun H, Sun L, Wu Q, Luo Y, Qi Q, Li H, et al. Lipidomic Signatures of Dairy Consumption and Associated Changes in Blood Pressure and Other Cardiovascular Risk Factors Among Chinese Adults. Hypertension. 2022;79(8):1617-28.

23. Zhang X-Z, Zheng S-X, Hou Y-M. A Non-Targeted Liquid Chromatographic-Mass Spectrometric Metabolomics Approach for Association with Coronary Artery Disease: An Identification of Biomarkers for Depiction of Underlying Biological Mechanisms. Medical Science Monitor. 2017;23:613-22.

24. Zhang X, Xu H, Li GH, Long MT, Cheung CL, Vasan RS, et al. Metabolomics Insights into Osteoporosis Through Association With Bone Mineral Density. Journal of Bone & Mineral Research. 2021;36(4):729-38.

25. Zhuang Z, Gao M, Yang R, Liu Z, Cao W, Huang T. Causal relationships between gut metabolites and Alzheimer's disease: a bidirectional Mendelian randomization study. Neurobiology of Aging. 2021;100:119.e15-.e18.

26. Zhuang Z, Li N, Wang J, Yang R, Wang W, Liu Z, et al. GWAS-associated bacteria and their metabolites appear to be causally related to the development of inflammatory bowel disease. European Journal of Clinical Nutrition. 2022;76(7):1024-30.
